# One-pot synthesis, characterization and antiviral properties of new benzenesulfonamide-based spirothiazolidinones

**DOI:** 10.1007/s11030-024-10912-x

**Published:** 2024-06-27

**Authors:** Çağla Begüm Apaydın, Lieve Naesens, Gökçe Cihan-Üstündağ

**Affiliations:** 1 Department of Pharmaceutical Chemistry, Faculty of Pharmacy, Istanbul University, Fatih, 34126 Istanbul, Turkey; 2 Department of Microbiology, Immunology and Transplantation, Rega Institute, KU Leuven, B-3000 Louvain, Belgium

**Keywords:** Synthesis, Antiviral activity, Sulfonamide, Spirothiazolidinone, Influenza virus

## Abstract

A novel series of benzenesulfonamide substituted spirothiazolidinone derivatives (**3a–j**) were synthesized, characterized and evaluated for their antiviral activity. The spirocyclic compounds were prepared by the condensation of 4-(aminosulfonyl)-2-methoxybenzohydrazide, appropriate cyclic ketones and 2-mercaptopropionic acid in a one-pot reaction. The structures of the new compounds were established by IR, ^1^H NMR, ^13^C NMR (APT), and elemental analysis. The new compounds were evaluated in vitro antiviral activity against influenza A/H1N1, A/H3N2 and B viruses, as well as herpes simplex virus type 1 (HSV-1), respiratory syncytial virus (RSV) and yellow fever virus (YFV). Two derivatives bearing propyl (**3d)** and *tert-*butyl (**3e)** substituents at position 8 of the spiro ring exhibited activity against influenza A/H1N1 virus with EC50 values in the range of 35–45 µM and no cytotoxicity at 100 μM, the highest concentration tested.

**Supplementary Information:**

The online version contains supplementary material available at 10.1007/s11030-024-10912-x.

## Introduction

Influenza A and B viruses are highly contagious human respiratory pathogens causing annual epidemics with high medical and socioeconomical burden [1]. Influenza A viruses are classified into different A/HxNx subtypes on the basis of two surface glycoproteins, hemagglutinin (HA; 18 known subtypes) and neuraminidase (NA; 11 known subtypes) [2]. The current strains of seasonal influenza A virus belong to subtype A/H1N1 [specifically, A(H1N1)pdm09, that entered the human population during the 2009 pandemic] and A/H3N2. For influenza B virus, the strains are divided in two phylogenetic *lineages* named B/Victoria and B/Yamagata [3]. Within the viral replication cycle, HA is required for virus attachment and entry, while NA mediates release of the virus as well as its penetration through mucus [4].

Since the current seasonal influenza vaccines have varying effectiveness [5], antiviral drugs are an essential complement for influenza prevention, treatment and pandemic preparedness [6]. As of today, three drug classes are approved. The adamantane derivatives (amantadine and rimantadine) block the M2 ion channel of influenza A virus but are no longer recommended for clinical use, due to widespread viral resistance against these inhibitors [7]. The neuraminidase inhibitors oseltamivir, zanamivir, laninamivir and peramivir prevent the release of progeny virions from infected cells. In many countries, oseltamivir is the standard-of-care for *influenza A and B*, however resistance against this drug needs to be closely monitored [8]. In recent years, inhibitors of the viral polymerase complex have received major attention, with favipiravir and baloxavir marboxil already approved in several countries [9–12]. In Russia and China, the broad-spectrum antiviral drug arbidol (also known as umifenovir) has been available since many years [13, 14]. This molecule is the only approved inhibitor of influenza virus entry. Besides other potential mechanisms [15], arbidol acts by preventing the conformational change of the HA trimer at low pH [16–18]. After the virus has entered by endocytosis, HA refolding is required to release the fusion peptide and trigger fusion of the viral and endosomal membranes [19]. The literature contains numerous small molecule inhibitors of HA refolding [reviewed in: [20, 21]], however their subtype-dependent or group-specific [22] anti-influenza A virus activity form a main obstacle for preclinical development.

**Fig. 1:**
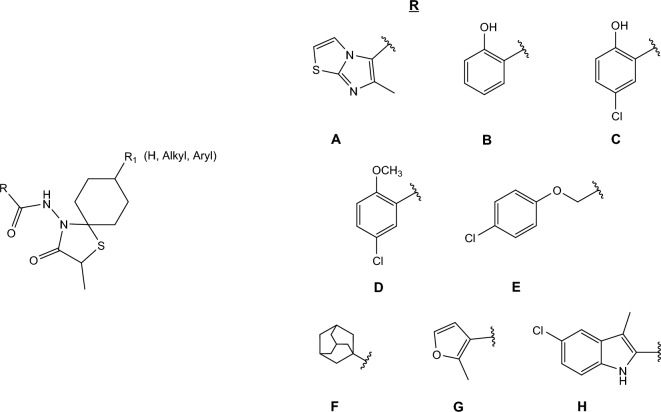
Chemical structures of previously reported influenza virus fusion inhibitors bearing the spirothiazolidinone scaffold

For over ten years now, research efforts in our laboratory have been focused on the synthesis and antiviral evaluation of compounds containing a spirocyclic ring system. In 2010, we identified a series of influenza virus fusion inhibitors with spirothiazolidinone (1-thia-4-azaspiro[4.5]decane) scaffold and strong cell culture activity against influenza A/H3N2 virus. Mechanistic studies established that these spirothiazolidinone compounds prevent the conformational change of H3 HA at low pH [23, 24]. These inhibitors share a common framework, consisting of an aromatic ring linked to a spirothiazolidinone system via an amide bridge. The lead compound **A**, identified in 2010, bears an imidazo[2,1-*b*]thiazole scaffold as the aromatic part (Fig. 1) [23]. Subsequent structure–activity relationship (SAR) studies demonstrated that the anti-A/H3N2 activity was maintained when the aromatic part was replaced by a substituted phenyl group (Fig. 1), i.e. *o*-hydroxyphenyl (**B**) [23], 5-chloro-2-hydroxyphenyl (**C**) [25], 5-chloro-2-methoxyphenyl (**D**) [26], 4-chlorophenoxymethyl (**E**) [27] or 1-adamantyl (**F**) [24], 2-methylfuran-3-yl (**G**) [28], 5-chloro-3-methyl-indole-2-yl (**H**) [18]. To date, all these spirothiazolidinone compounds exhibit narrow activity against influenza A/H3 HA, with no inhibition of A/H1, A/H5 and A/H7 nor of influenza B HA [23].

Based on our previous biological results, we designed a new series of benzamide-derived spiro compounds with a methoxy substituent at *ortho* position and an electron-withdrawing sulfonamide group at *para* position. The sulfonamide group is important in medicinal chemistry, since a wide variety of drugs have the benzene sulfonamide nucleus. Antiviral activity is also observed in a wide range of compounds with a sulfonamide fragment in combination with aromatic or heteroaromatic rings [29, 30]. Therefore, we decided to examine the influence of introducing the sulfonamide moiety. We here report the chemical synthesis, structural characterization and antiviral evaluation of this series of 2-methoxy-*N*-(2-methyl-3-oxo-1-thia-4-azaspiro[4.5]decan-4-yl)-4-sulfamoylbenzamides (**3a–j**) against influenza A/H1N1, A/H3N2 and B viruses, as well as herpes simplex virus type 1 (HSV-1), respiratory syncytial virus (RSV) and yellow fever virus (YFV).

## Results and discussion

### Chemistry

**Scheme 1:**
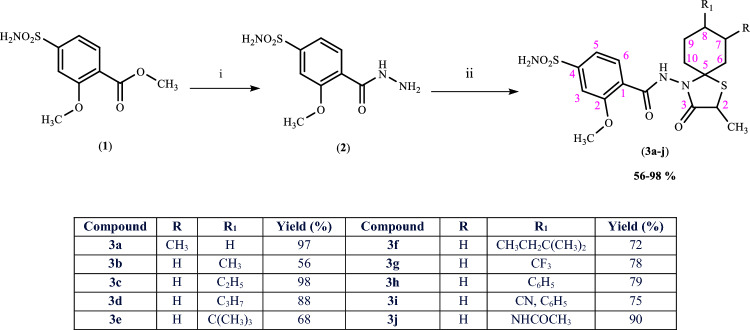
Synthesis of compounds **3a–j**. Reagents and conditions: (i) hydrazine hydrate, ethanol, reflux, 2 h; (ii) substituted ketone, 2-sulfanylpropanoic acid, toluene, 6–8 h

The synthetic pathway for the preparation of new spirothiazolidinones (**3a–j**) is demonstrated in Scheme 1. Three experimental approaches have been described in the literature for spirothiazolidinone cyclization: traditional two-step method [31, 32], greener one-pot method [23, 27, 28] and microwave-assisted green one-pot synthesis [24, 26]. In this study, new spirocyclic compounds were obtained by one-pot reaction. Thus, the key intermediate 4-(aminosulfonyl)-2-methoxybenzohydrazide (**2**) was reacted with an appropriate cyclic ketone and 2-mercaptopropionic acid in one-pot, using a Dean Stark water separator. The novel compounds (**3a–j)** were characterized by combustion analysis and IR, ^1^H NMR and ^13^C NMR (APT) spectral studies.

The detailed spectral data of compounds **3a–j** are shown in the experimental section. The solid phase (KBr) IR spectra of **3a–j** showed common characteristic absorption bands at 3336–3211 cm^−1^ (N–H stretching bands), 1699–1685 cm^−1^ (lactam C=O bands) and 1674–1639 cm^−1^ (amide C=O bands), which provided evidence for the cycloaddition reaction. The ^1^H NMR spectra of the synthesized compounds showed characteristic broad singlets of benzamide NH group at *δ* 10.54–10.26 ppm. The S-CH protons of the newly formed thiazolidinone residue resonated as quartets at *δ* 4.04–3.90 ppm, confirming the structure of the desired compounds. The remaining proton signals of spiroalkane system were detected at *δ* 3.48–0.68 ppm region, together with the alkyl substituents. The resonances of 2-OCH_3_ and 4-SO_2_NH_2_ groups at the phenyl subunit were observed as singlets in the *δ* 3.98–3.92 ppm and δ 7.39–7.34 ppm, respectively. Peaks associated with the aromatic ring were observed in the expected regions (*δ* 8.06–7.32 ppm) and the splitting patterns were in accordance with the 1,2,4-trisubstituted aromatic ring system. APT spectra of **3a–j** showed two downfield signals at about δ 170.5–164.6 ppm due to the carbonyl carbon absorptions. Observation of upfield resonances assigned to the aliphatic CH/CH_2_ carbons and the typical spirodecan C5 resonances (*δ* 71.6–69.5 ppm) substantiated the formation of the expected spirothiazolidinones.

### Antiviral activity

The anti-influenza virus activity of the ten new spirocyclic compounds was determined in Madin-Darby canine kidney (MDCK) cells, using two strains of influenza A virus [A/Virginia/ATCC3/2009 (A/H1N1) and A/HK/7/87 (A/H3N2)] and one strain of influenza B virus [B/Ned/537/05]. In addition, the compounds were evaluated against HSV-1 and RSV in HEL299 cells and against YFV in Huh7 cells. The 50% effective concentration (EC_50_) was defined as the compound concentration producing 50% inhibition of virus-induced cytopathic effect (CPE), as assessed by microscopic scoring and MTS cell viability assay. In parallel, compound cytotoxicity was determined in mock-infected cultures, and expressed as minimal cytotoxic concentration (MCC, based on microscopy) and 50% cytotoxic concentration (CC_50_, by MTS assay) (Table 1).

**Table 1:** Antiviral activity in MDCK^a^ cells infected with influenza A or B virus

Compound	Antiviral EC_50_^b^ (µM)						Cytotoxicity (µM)	
	Influenza A/H1N1		Influenza A/H3N2		Influenza B			
	Microscopy	MTS	Microscopy	MTS	Microscopy	MTS^c^	MCC^d^	CC_50_^e^
**3a**	> 100	76	> 100	64	> 100	ND	> 100	> 100
**3b**	> 100	> 100	> 100	> 100	> 100	ND	> 100	> 100
**3c**	> 100	80	> 100	> 100	> 100	ND	100	> 100
**3d**	45	39	> 100	> 100	> 100	ND	100	> 100
**3e**	45	35	> 100	68	> 100	ND	> 100	> 100
**3f**	> 100	> 100	> 100	> 100	> 100	ND	> 100	> 100
**3 g**	> 100	> 100	> 100	> 100	> 100	ND	100	> 100
**3 h**	> 100	> 100	> 100	> 100	> 100	ND	100	> 100
**3i**	> 100	74	> 100	> 100	> 100	ND	> 100	> 100
**3j**	> 100	> 100	> 100	> 100	> 100	ND	> 100	> 100
Ribavirin	45	25	40	26	10	ND	100	> 100
Zanamivir	> 100	> 100	< 0.8	< 0.8	< 0.8	ND	> 100	> 100
BXA^f^ (nM)	1.8	1.5	2.0	1.7	8.3	ND	> 20	> 20

^a^Madin Darby canine kidney cells

^b^EC_50_: compound concentration producing 50% inhibition of virus-induced cytopathic effect, as determined by microscopic scoring or by MTS cell viability assay

^c^*ND* Not determined

For influenza B virus, the MTS-OD values between virus control and cell control were not sufficiently different to reliably determine the compounds’ activity by this method

^d^Minimal compound concentration that causes a microscopically detectable alteration of normal cell morphology

^e^50% cytotoxic concentration based on MTS cell viability assay

^f^*BXA* baloxavir acid (concentration unit: nM)

Since our previous studies indicate that the anti-A/H3N2 activity of the spirothiazolidinone compounds is preserved for several variations of the aromatic part (see above), it is quite surprising that neither of the new compounds had noticeable activity against A/H3N2 virus (Table 1). They all carry a methyl group at position 2 in the spiro ring, which we proved to be a crucial substituent [18, 23]. Also, the new compounds **3b** (8-methyl) and **3c** (8-ethyl) are the direct analogues of the highly potent and published compounds **4c** and **4d** [23] and **5e** and **5f** [18], the only difference being the structure of the aromatic part. On the other hand, two of the new compounds displayed weak activity against A/H1N1 virus, with compound **3d** (8-propyl) and **3e** (8-*tert*-butyl) having EC_50_ values in the range of 35–45 µM and no cytotoxicity at 100 μM, the highest concentration tested. At this concentration, neither of the compounds had an effect on the replication of HSV-1, RSV or YFV (data not shown).

## Conclusion

A series of novel spirothiazolidinone compounds carrying 2-methoxy-4-sulfamoylbenzamide moiety (**3a–j**) have been synthesized, characterized, and evaluated as replication inhibitors of influenza virus. Two compounds (**3d** and **3e**) displayed weak activity against influenza A/H1N1 virus. This is unexpected since, so far, our spirothiazolidinone class of fusion inhibitors was active against A/H3N2 but not A/H1N1 virus. This includes the previously synthesized *o*-hydroxy and *o*-methoxy substituted phenyl derivatives (**B, C** and **D** in Fig. 1). Most likely, the electron-withdrawing sulfonamide group on the *para* position of the benzene ring is responsible for the change in anti-influenza virus activity profile. On the other hand, our new data indicate that the inhibitory activity of the spirothiazolidinone class is amenable towards specific HA subtypes, with even subtle differences having the ability to change the binding properties in the HA binding pocket [18]. Hence, we have embarked on further structural optimization of the aromatic part, to conduct mechanistic antiviral experiments and hopefully increase the anti-A/H1N1 activity.

## Experimental section

### Materials

Chemicals were obtained from Sigma Aldrich. Reaction progress was monitored by thin layer chromatography (TLC) using silica gel plates and chloroform:methanol (9:1) as the eluent. Melting points (mp) were determined on a Buchi B-540 capillary melting point apparatus in open capillaries and uncorrected. IR spectra were recorded in KBr discs on a Shimadzu IR Affinity-1 FTIR. ^1^H NMR (DMSO-d_6_) spectra were run on a Varian^MERCURY^400 MHZ and ^13^C NMR (APT) (DMSO-d_6_) spectra were run Bruker 500 MHz spectrophotometers. Microanalyses were performed on a Thermo Finnigan Flash EA 1112 elemental analyzer. (Sp: spirothiazolidinone, Ar: aromatic ring).

### Chemical synthesis

#### General procedure for the synthesis of compound 4-(aminosulfonyl)-2-methoxybenzohydrazide (2)

To the solution of 0.05 mol of methyl 2-methoxy-4-sulfamoylbenzoate (**1)** in 10 mL of ethanol was added 0.1 mol of 99% hydrazine hydrate. The mixture was refluxed for 2 h. The reaction mixture was then cooled, diluted with water and allowed to stand overnight and used as a crude product. *R*_f_ (**2**) = 0.22.

#### General procedure for the synthesis of compounds 3a-j

A mixture of **2** (0.005 mol) and appropriate ketone (0.01 mol) in 30 mL of dried toluene were refluxed for 1 h, using a Dean Stark water separator. After 1 h, 2-sulfanylpropanoic acid (0.01 mol) was added and the mixture was refluxed during 6–8 h. Excess toluene was evaporated in vacuo. The resulting residue was treated with saturated NaHCO_3_ solution until CO_2_ evolution ceased and was allowed to stand overnight or in some cases refrigerated until solidification. The precipitate was filtered and purified by recrystallization from ethanol.

#### 2-Methoxy-*N*-(2,7-dimethyl-3-oxo-1-*thia*-4-azaspiro[4.5]decan-4-yl)-4-sulfamoylbenzamide (3a)

White powder (97%); R_*f*_ (**3a**) = 0.57; m.p: 261–264 °C; IR (KBr): *υ*_max_ 3294, 3211 (N–H), 1693 (C=O), 1662 (NHC=O), 1342, 1168 (S=O). ^1^H NMR (DMSO-d_6_/400 MHz): *δ* 10.26 (1H, s, NH), 7.99, 8.00 (1H, 2d, *J* = 2.4 Hz, Ar-H3), 7.91 (1H, dd, *J* = 8.8, 2.4 Hz, Ar-H5), 7.34 (2H, s, SO_2_NH_2_), 7.32 (1H, d, *J* = 8.9 Hz, Ar-H6), 3.93 (3H, s, OCH_3_), 3.91 (1H, q, *J* = 7.0 Hz, Sp-S-CH), 1.91–1.46 (8H, m, Sp-CH/CH_2_), 1.43 (3H, d, *J* = 6.8 Hz, Sp-2-CH_3_), 0.89 (3H, d, *J* = 6.0 Hz, Sp-7-CH_3_), 0.80–0.68 (1H, m, Sp-CH/CH_2_). ^13^C NMR (DMSO-d_6_/125 MHz): 170.4 (Sp-CO), 164.7 (NHCO), 159.4 (Ar-C2), 136.6 (Ar-C4), 130.6, 130.5, 128.3, 128.2 (Ar-C5,C6), 123.2, 123.1 (Ar-C1), 112.8 (Ar-C3), 71.4 (Ar-C5), 57.1, 57.0 (OCH_3_), 46.8, 46.5 (Sp-CH_2_), 37.9, 36.9, 33.4, (Sp-CH_2_), 37.3, 37.2 (Sp-C2), 30.3, 29.8 (Sp-C7), 23.20 (Sp-CH_2_), 22.7, 22.5 (Sp-7-CH_3_), 20.2, 19.9 (Sp-2-CH_3_). Anal. calcd. for C_18_H_25_N_3_O_5_S_2_ (427.53) C: 50.57, H: 5.89, N: 9.83. Found C: 50.52, H:6.20, N: 9.75.

#### 2-Methoxy-*N*-(2,8-dimethyl-3-oxo-1-thia-4-azaspiro[4.5]decan-4-yl)-4-sulfamoylbenzamide (3b)

White powder (56%); *R*_*f*_ (**3b**) = 0.64; m.p: 258–261 °C; IR (KBr): *υ*_max_ 3321, 3219 (N–H), 1691 (C=O), 1641 (NHC=O), 1323, 1159 (S=O). ^1^H NMR (DMSO-d_6_/400 MHz): *δ* 10.32 (1H, s, NH), 8.03 (1H, d, *J* = 2.4 Hz, Ar-H3), 7.95 (1H, dd, *J* = 8.8, 2.4 Hz, Ar-H5), 7.39 (2H, s, SO_2_NH_2_), 7.35 (1H, d, *J* = 8.9 Hz, Ar-H6), 3.96 (3H, s, OCH_3_), 3.94 (1H, q, *J* = 7.0 Hz, Sp-S-CH), 2.08–1.70 (6H, m, Sp-CH/CH_2_), 1.46 (3H, d, *J* = 7.0 Hz, Sp-2-CH_3_), 1.36–1.07 (3H, m, Sp-CH/CH_2_), 0.90 (3H, d, *J* = 6.2 Hz, Sp-8-CH_3_). ^13^C NMR (DMSO-d_6_/125 MHz): 170.5 (Sp-CO), 164.7 (NHCO), 159.5 (Ar-C2), 136.6 (Ar-C4), 130.5, 128.2 (Ar-C5, C6), 123.1 (Ar-C1), 112.8 (Ar-C3), 71.3 (Ar-C5), 57.0 (OCH_3_), 38.1 (Sp-CH_2_), 37.2 (Sp-C2), 32.1, 31.7 (Sp-CH_2_), 31.2 (Sp-C8), 22.3 (Sp-8-CH_3_), 20.1 (Sp-2-CH_3_). Anal. calcd. for C_18_H_25_N_3_O_5_S_2_ (427.53) C: 50.57, H: 5.89, N: 9.83. Found C: 50.57, H: 5.86, N: 9.88.

#### 2-Methoxy-*N*-(8-ethyl-2-methyl-3-oxo-1-thia-4-azaspiro[4.5]decan-4-yl)-4-sulfamoylbenzamide (3c)

White powder (98%); *R*_*f*_ (**3c**) = 0.40; m.p: 270–272 °C; IR (KBr): *υ*_max_ 3317, 3219 (N–H), 1689 (C=O), 1645 (NHC=O), 1321, 1159 (S=O). ^1^H NMR (DMSO-d_6_/400 MHz): *δ* 10.27 (1H, s, NH), 8.00 (1H, d, *J* = 2.4 Hz, Ar-H3), 7.91 (1H, dd, *J* = 8.8, 2.4 Hz, Ar-H5), 7.34 (2H, s, SO_2_NH_2_), 7.32 (1H, d, *J* = 8.8 Hz, Ar-H6), 3.93 (3H, s, OCH_3_), 3.90 (1H, q, *J* = 7.0 Hz, Sp-S-CH), 2.03–1.69 (6H, m, Sp-CH/CH_2_), 1.42 (3H, d, *J* = 6.8 Hz, Sp-2-CH_3_), 1.27–1.00 (5H, m, Sp-CH/CH_2_, 8-CH_2_CH_3_), 0.84 (3H, t, *J* = 7.4 Hz, Sp-8-CH_2_CH_3_). ^13^C NMR (DMSO-d_6_/125 MHz): 170.4 (Sp-CO), 164.7 (NHCO), 159.5 (Ar-C2), 136.6 (Ar-C4), 130.5, 128.2 (Ar-C5, C6), 123.1 (Ar-C1), 112.8 (Ar-C3), 71.6 (Ar-C5), 57.0 (OCH_3_), 38.1, 29.6, 29.2 (Sp-CH_2_, Sp-8-CH_2_CH_3_), 37.7, 37.2 (Sp-C2, C8), 20.1 (Sp-2-CH_3_), 11.8 (Sp-8-CH_2_CH_3_). Anal. calcd. for C_19_H_27_N_3_O_5_S_2_ (441.56) C: 51.68, H: 6.16, N: 9.52. Found C: 51.29, H: 6.26, N: 9.46.

#### 2-Methoxy-*N*-(2-methyl-3-oxo-8-propyl-1-thia-4-azaspiro[4.5]decan-4-yl)-4-sulfamoylbenzamide (3d)

White powder (88%); R_*f*_ (**3d**) = 0.44; m.p: 274–276 °C; IR (KBr): *υ*_max_ 3315, 3221 (N–H), 1689 (C=O), 1645 (NHC=O), 1319, 1159 (S=O). ^1^H NMR (DMSO-d_6_/400 MHz): δ 10.30 (1H, s, NH), 8.00 (1H, d, *J* = 2.4 Hz, Ar-H3), 7.91 (1H, dd, *J* = 8.7, 2.5 Hz, Ar-H5), 7.36 (2H, s, SO_2_NH_2_), 7.32 (1H, d, *J* = 8.8 Hz, Ar-H6), 3.92 (3H, s, OCH_3_), 3.90 (1H, q, *J* = 7.0 Hz, Sp-S-CH), 2.03–1.68 (6H, m, Sp- CH/CH_2_), 1.42 (3H, d, *J* = 7.0 Hz, Sp-2-CH_3_), 1.34–0.98 (7H, m, Sp-CH/CH_2_, 8-CH_2_CH_2_CH_3_), 0.84 (3H, t, *J* = 7.2 Hz, Sp-8-CH_2_CH_2_CH_3_). ^13^C NMR (DMSO-d_6_/125 MHz): 170.5 (Sp-CO), 164.7 (NHCO), 159.5 (Ar-C2), 136.6 (Ar-C4), 130.5, 128.3 (Ar-C5, C6), 123.1 (Ar-C1), 112.8 (Ar-C3), 71.6 (Sp-C5), 56.9 (OCH_3_), 38.8, 38.1, 30.1, 29.6 (Sp-CH_2_, Sp-8-CH_2_CH_2_CH_3_), 37.2 (Sp-C2), 35.7 (Sp-C8), 20.1 (Sp-2-CH_3_), 19.9 (Sp-8-CH_2_CH_2_CH_3_), 14.6 (Sp-8-CH_2_CH_2_CH_3_). Anal. calcd. for C_20_H_29_N_3_O_5_S_2_ (455.59) C: 52.73, H: 6.42, N: 9.22. Found C: 52.58, H: 6.65, N: 9.30.

#### 2-Methoxy-*N*-(2-methyl-3-oxo-8-*tert*-butyl-1-thia-4-azaspiro[4.5]decan-4-yl)-4-sulfamoylbenzamide (3e)

White powder (68%); R_*f*_ (**3e**) = 0.46; m.p: 301–304 °C; IR (KBr): υ_max_ 3336, 3302, 3219 (N–H), 1691 (C=O), 1651 (NHC=O), 1323, 1159 (S=O). ^1^H NMR (DMSO-d_6_/400 MHz): δ 10.26 (1H, s, NH), 8.02 (1H, d, *J* = 2.4 Hz, Ar-H3), 7.92 (1H, dd, *J* = 8.7, 2.5 Hz, Ar-H5), 7.34 (2H, s, SO_2_NH_2_), 7.32 (1H, d, *J* = 8.9 Hz, Ar-H6), 3.93 (3H, s, OCH_3_), 3.90 (1H, q, *J* = 7.0 Hz, Sp-S-CH), 2.01–1.71 (6H, m, Sp-CH/CH_2_), 1.43 (3H, d, *J* = 7.0 Hz, Sp-2-CH_3_), 1.31–1.11 (2H, m, Sp-CH/CH_2_), 0.96–0.87 (1H, m, Sp-CH/CH_2_), 0.83 (9H, s, Sp-8-C(CH_3_)_3_). ^13^C NMR (DMSO-d_6_/125 MHz): 170.5 (Sp-CO), 164.7 (NHCO), 159.5 (Ar-C2), 136.6 (Ar-C4), 130.6, 128.3 (Ar-C5, C6), 123.0 (Ar-C1), 112.8 (Ar-C3), 71.5 (Sp-C5), 57.0 (OCH_3_), 46.4 (Sp-C8), 38.4, 37.6, 24.5, 24.0 (Sp-CH_2_), 37.2 (Sp-C2), 32.4 (Sp-8-C(CH_3_)_3_), 27.7 (Sp-8-C(CH_3_)_3_), 20.1 (Sp-2-CH_3_). Anal. calcd. for C_21_H_31_N_3_O_5_S_2_ (469.61) C: 53.71, H: 6.65, N: 8.95. Found C: 53.73, H: 6.74, N: 9.11.

#### 2-Methoxy-*N*-(2-methyl-3-oxo-8-*tert*-pentyl-1-thia-4-azaspiro[4.5]decan-4-yl)-4-sulfamoylbenzamide (3f)

White powder (72%); R_*f*_ (**3f**) = 0.67; m.p: 290–295 °C; IR (KBr): *υ*_max_ 3321, 3223 (N–H), 1691 (C=O), 1651 (NHC=O), 1328, 1159 (S=O). ^1^H NMR (DMSO-d_6_/400 MHz): *δ* 10.28 (1H, s, NH), 8.01 (1H, d, *J* = 2.4 Hz, Ar-H3), 7.92 (1H, dd, *J* = 8.8, 2.5 Hz, Ar-H5), 7.36 (2H, s, SO_2_NH_2_), 7.32 (1H, d, *J* = 8.8 Hz, Ar-H6), 3.92 (3H, s, OCH_3_), 3.90 (1H, q, *J* = 7.0 Hz, Sp-S-CH), 2.02–1.64 (6H, m, Sp-CH/CH_2_), 1.42 (3H, d, *J* = 7.0 Hz, Sp-2-CH_3_), 1.33–1.11 (2H, m, Sp-CH/CH_2_), 1.22 (2H, q, *J* = 7.6 Hz, Sp-8-C(CH_3_)_2_CH_2_CH_3_), 1.04–0.92 (1H, m, Sp-CH/CH_2_), 0.77 (6H, s, Sp-8-C(CH_3_)_2_CH_2_CH_3_), 0.70 (3H, t, *J* = 7.6 Hz, Sp-8-C(CH_3_)_2_CH_2_CH_3_). ^13^C NMR (DMSO-d_6_/125 MHz): 170.5 (Sp-CO), 164.7 (NHCO), 159.5 (Ar-C2), 136.7 (Ar-C4), 130.5, 128.3 (Ar-C5, C6), 123.0 (Ar-C1), 112.8 (Ar-C3), 71.5 (Sp-C5), 43.7 (Sp-C8), 38.5, 37.6 (Sp-CH_2_), 37.2 (Sp-C2), 34.6, 32.6 (Sp-8-C(CH_3_)_2_CH_2_CH_3_), 24.53 (Sp-8-C(CH_3_)_2_CH_2_CH_3_), 24.0, 23.6 (Sp-CH_2_), 20.1 (Sp-2-CH_3_), 8.4 (Sp-8-C(CH_3_)_2_CH_2_CH_3_). Anal. calcd. for C_22_H_33_N_3_O_5_S_2_ (483.64) C: 54.63, H: 6.88, N: 8.69. Found C: 54.94, H: 7.29, N: 8.70.

#### 2-Methoxy-*N*-(2-methyl-3-oxo-1-thia-8-trifluoromethyl-4-azaspiro[4.5]decan-4-yl)-4-sulfamoylbenzamide (3 g)

White powder (78%); *R*_*f*_ (**3 g**) = 0.45; m.p: 210–214 °C; IR (KBr): *υ*_max_ 3311, 3221 (N–H), 1689 (C=O), 1647 (NHC=O), 1390, 1159 (S=O). ^1^H NMR (DMSO-d_6_/400 MHz): δ 10.31 (1H, s, NH), 8.06 (1H, d, *J* = 2.4 Hz, Ar-H3), 7.93 (1H, dd, *J* = 8.8, 2.5 Hz, Ar-H5), 7.36 (2H, s, SO_2_NH_2_), 7.33 (1H, d, *J* = 8.9 Hz, Ar-H6), 3.97 (1H, q, *J* = 7.0 Hz, Sp-S-CH), 3.95 (3H, s, OCH_3_), 2.38–2.18 (1H, m, Sp-CH/CH_2_), 2.11–1.70 (6H, m, Sp-CH/CH_2_), 1.62–1.36 (2H, m, Sp-CH/CH_2_), 1.43 (3H, d, *J* = 7.0 Hz, Sp-2-CH_3_). ^13^C NMR (DMSO-d_6_/125 MHz): 170.4 (Sp-CO), 164.7 (NHCO), 159.6 (Ar-C2), 136.6 (Ar-C4), 130.8, 128.5 (Ar-C5, C6), 128.2 (q, *J* = 277.0 Hz, Sp-8-CF_3_), 122.6 (Ar-C1), 112.9 (Ar-C3), 70.3 (Sp-C5), 57.1 (OCH_3_), 39.0 (d, *J* = 26 Hz, Sp-C8), 37.3 (Sp-C2), 36.3, 35.5, 22.4, 21.9 (Sp-CH_2_), 19.9 (Sp-2-CH_3_). Anal. calcd. for C_18_H_22_F_3_N_3_O_5_S_2_ (481.50) C: 44.90, H: 4.61, N: 8.73. Found C: 44.69, H: 4.60, N: 8.82.

#### 2-Methoxy-*N*-(2-methyl-3-oxo-8-phenyl-1-thia-4-azaspiro[4.5]decan-4-yl)-4-sulfamoylbenzamide (3 h)

White powder (79%); *R*_*f*_ (**3 h**) = 0.56; m.p: 290–292 °C; IR (KBr): *υ*_max_ 3304, 3217 (N–H), 1685 (C=O), 1639 (NHC=O), 1319, 1159 (S=O). ^1^H NMR (DMSO-d_6_/400 MHz): *δ* 10.35 (1H, s, NH), 8.06 (1H, d, *J* = 2.4 Hz, Ar-H3), 7.94 (1H, dd, *J* = 8.8, 2.5 Hz, Ar-H5), 7.36 (2H, s, SO_2_NH_2_), 7.35 (1H, d, *J* = 8.9 Hz, Ar-H6), 7.31–7.14 (5H, m, Sp-8-C_6_H_5_), 3.98 (3H, s, OCH_3_), 3.96 (1H, q, *J* = 7.0 Hz, Sp-S-CH), 2.50–2.42 (m, DMSO-d_6_ and Sp-C8-H), 2.23–1.84 (6H, m, Sp-CH/CH_2_), 1.78–1.55 (2H, m, Sp-CH/CH_2_), 1.46 (3H, d, *J* = 7.0 Hz, Sp-2-CH_3_). ^13^C NMR (DMSO-d_6_/125 MHz): 170.5 (Sp-CO), 164.8 (NHCO), 159.6 (Ar-C2), 146.2 (Sp-8-C_6_H_5_(C1)), 136.7 (Ar-C4), 130.6, 128.4 (Ar-C5, C6), 128.4, 127.2, 126.6 (Sp-8-C_6_H_5_(C2-6)), 122.9 (Ar-C1), 112.9 (Ar-C3), 71.0 (Sp-C5), 57.1 (OCH_3_), 42.1 (Sp-C8), 38.4, 37.5 (Sp-CH_2_), 37.3 (Sp-C2), 31.3, 30.7 (Sp-CH_2_), 20.1 (Sp-2-CH_3_). Anal. calcd. for C_23_H_27_N_3_O_5_S_2_ (489.60) C: 56.42, H: 5.56, N: 8.58. Found C: 56.34, H: 5.70, N: 8.59.

#### 2-Methoxy-*N*-(8-cyano-2-methyl-3-oxo-8-phenyl-1-thia-4-azaspiro[4.5]decan-4-yl)-4-sulfamoylbenzamide (3i)

White powder (75%); *R*_*f*_ (**3i**) = 0.36; m.p: 265–270 °C; IR (KBr): *υ*_max_ 3261 (N–H), 1699 (C=O), 1672 (NHC=O), 1334, 1165 (S=O). ^1^H NMR (DMSO-d_6_/400 MHz): *δ* 10.54 (1H, s, NH), 7.98 (1H, d, *J* = 2.5 Hz, Ar-H3), 7.93 (1H, dd, *J* = 8.7, 2.5 Hz, Ar-H5), 7.53 (2H, d, *J* = 7.6 Hz, 8-C_6_H_5_(H2,H6), 7.45 (2H, t, *J* = 7.6 Hz, 8-C_6_H_5_(H3,H5), 7.40–7.33 (4H, m, Sp-8-C_6_H_5_(H4), SO_2_NH_2_ and Ar-H6), 4.04 (1H, q, *J* = 7.0 Hz, Sp-S-CH), 3.97 (3H, s, OCH_3_), 2.60–2.33 (m, DMSO-d_6_ and Sp-CH_2_), 2.14–1.93 (4H, m, Sp-CH_2_), 1.47 (3H, d, *J* = 7.0 Hz, Sp-2-CH_3_). ^13^C NMR (DMSO-d_6_/125 MHz): 170.4 (Sp-CO), 165.1 (NHCO), 159.4 (Ar-C2), 140.2 (Sp-8-C_6_H_5_(C1)), 36.6 (Ar-C4), 130.5, 128.0 (Ar-C5,C6), 129.4, 128.7, 126.2 (Sp-8-C_6_H_5_(C2-6)), 122.2 (Ar -C1), 122.2 (Sp-4-CN), 112.7 (Ar-C3), 69.7 (Sp-C5), 57.0 (OCH_3_), 42.3 (Sp-C8), 37.4 (Sp-C2), 35.6, 34.9, 34.1, 33.5 (Sp-CH_2_), 20.0 (Sp-2-CH_3_). Anal. calcd. for C_24_H_26_N_4_O_5_S_2_.H_2_O (532.57) C: 54.12, H: 5.30, N: 10.89. Found C: 53.99, H: 5.11, N: 10.52.

#### 2-Methoxy-*N*-(8-acetamido-2-methyl-3-oxo-1-thia-4-azaspiro[4.5]decan-4-yl)-4-sulfamoylbenzamide (3j)

White powder (90%); *R*_*f*_ (**3j**) = 0.60; m.p: 220–223 °C; IR (KBr): *υ*_max_ 3269, 3228 (N–H), 1699 (C=O), 1674, 1622 (NHC=O), 1340, 1165 (S=O). ^1^H NMR (DMSO-d_6_/400 MHz): *δ* 10.34 (1H, s, NH), 8.00 (1H, d, *J* = 2.5 Hz, Ar-H3), 7.92 (1H, dd, *J* = 8.8, 2.5 Hz, Ar-H5), 7.83 (1H, d, *J* = 7.7 Hz, NHCOCH_3_), 7.36 (2H, s, SO_2_NH_2_), 7.33 (1H, d, *J* = 8.9 Hz, Ar-H6), 3.96 (1H, q, *J* = 7.0 Hz, Sp-S-CH), 3.94 (3H, s, OCH_3_), 3.48–3.36 (1H, m, Sp-C8-H), 2.13–1.76 (6H, m, Sp-CH_2_), 1.75 (3H, s, NHCOCH_3_), 1.51–1.29 (2H, m, sp-CH_2_), 1.43 (3H, d, *J* = 7.0 Hz, Sp-2-CH_3_). ^13^C NMR (DMSO-d_6_/125 MHz): 170.5 (Sp-CO), 164.9, 164.6 (NHCO, NHCOCH_3_), 159.5 (Ar-C2), 136.9, 136.7, 136.0 (Ar-C4), 130.0, 128.9, 127.2 (Ar-C5,C6), 123.1 (Ar-C1), 112.9 (Ar-C3), 69.5 (Sp-C5), 57.3 (OCH_3_), 37.2 (Sp-C2), 35.4 (Sp-C8), 29.4, 29.1 (Sp-CH_2_), 21.6 (Sp-8-NHCOCH_3_), 19.9 (Sp-2-CH_3_). Anal. calcd. for C_16_H_22_N_4_O_5_S. 1/2H_2_O (391.43) C: 49.10, H: 6.13, N: 14.32. Found C: 49.62, H: 5.89, N: 14.50.

### Antiviral procedures

The influenza virus cytopathic effect (CPE) reduction assay was reported in full detail elsewhere [33]. Briefly, Madin-Darby canine kidney (MDCK) cells were seeded in 96-well plates at 7,500 cells per well, using Ultra-MDCK medium (from Lonza) supplemented with 2 µg per ml of trypsin. On the next day, they were infected with 100 CCID_50_ (50% cell culture infective dose) per well of influenza A/H1N1 (A/Virginia/ATCC3/2009) or A/H3N2 (A/HK/7/87) virus (both from ATCC), or influenza B virus (B/Ned/537/05; kind gift from R. Fouchier). At the same time, the compounds were added at serial dilutions. Mock-infected plates prepared in parallel received the compounds but no virus. After four days incubation at 35 °C, the virus-induced CPE was scored by microscopy, after which the colorimetric MTS assay (CellTiter 96® AQueous One Solution Cell Proliferation Assay from Promega) was conducted. The same two methods were applied to the mock-infected plate, to determine compound cytotoxicity. Antiviral activity was defined as the 50% effective concentration (EC_50_), whereas cytotoxicity was expressed as MCC (minimal cytotoxic concentration, based on microscopy) or CC_50_ (50% cytotoxic concentration, assessed by the MTS assay) [see reference (28) for calculation methods].

Analogous CPE reduction assays were used for HSV-1 (strain KOS) and RSV (strain Long), both assessed in HEL299 human embryonic lung fibroblast cells, and for YFV (strain 17D), assessed in Huh-7 human liver carcinoma cells. After infection and compound addition, the cells were incubated for 3–6 days at 37 °C, until full-blown CPE was visible. The inhibitory effect on virus-induced CPE and compound cytotoxicity was determined by microscopy and MTS assay, and the data were analyzed as above.

## Supplementary Information

Below is the link to the electronic supplementary material.

Supplementary file1 (PDF 1651 KB)
