# Supplementary material for: One-pot synthesis, characterization and antiviral properties of new benzenesulfonamide-based spirothiazolidinones
Source: Mol Divers. 2024 Jun 27;28(4):2681–8. doi: 10.1007/s11030-024-10912-x (PMC11450120; doi:10.1007/s11030-024-10912-x)
Supplement: Supplementary file 1 — Supplementary file1 (PDF 1651 KB) [file 11030_2024_10912_MOESM1_ESM.pdf]

Synthesis, characterization and antiviral properties of new benzenesulfonamide-based spirothiazolidinones

Çağla Begüm Apaydın[1]*, Lieve Naesens[2], Gökçe Cihan-Üstündağ[1]

[1]Istanbul University, Faculty of Pharmacy, Department of Pharmaceutical Chemistry, Istanbul, Turkey

[2]Rega Institute, KU Leuven, Department of Microbiology, Immunology and Transplantation, B-3000, Leuven, Belgium

*Corresponding author: Çağla Begüm Apaydın, Faculty of Pharmacy, Department of Pharmaceutical Chemistry, Istanbul University, 34126, Istanbul, Fatih, Turkey.
E-mail: cagla.apaydin@istanbul.edu.tr

2-Methoxy-*N*-(2,7-dimethyl-3-oxo-1-thia-4-azaspiro[4.5]decan-4-yl)-4-sulfamoylbenzamide (3a)

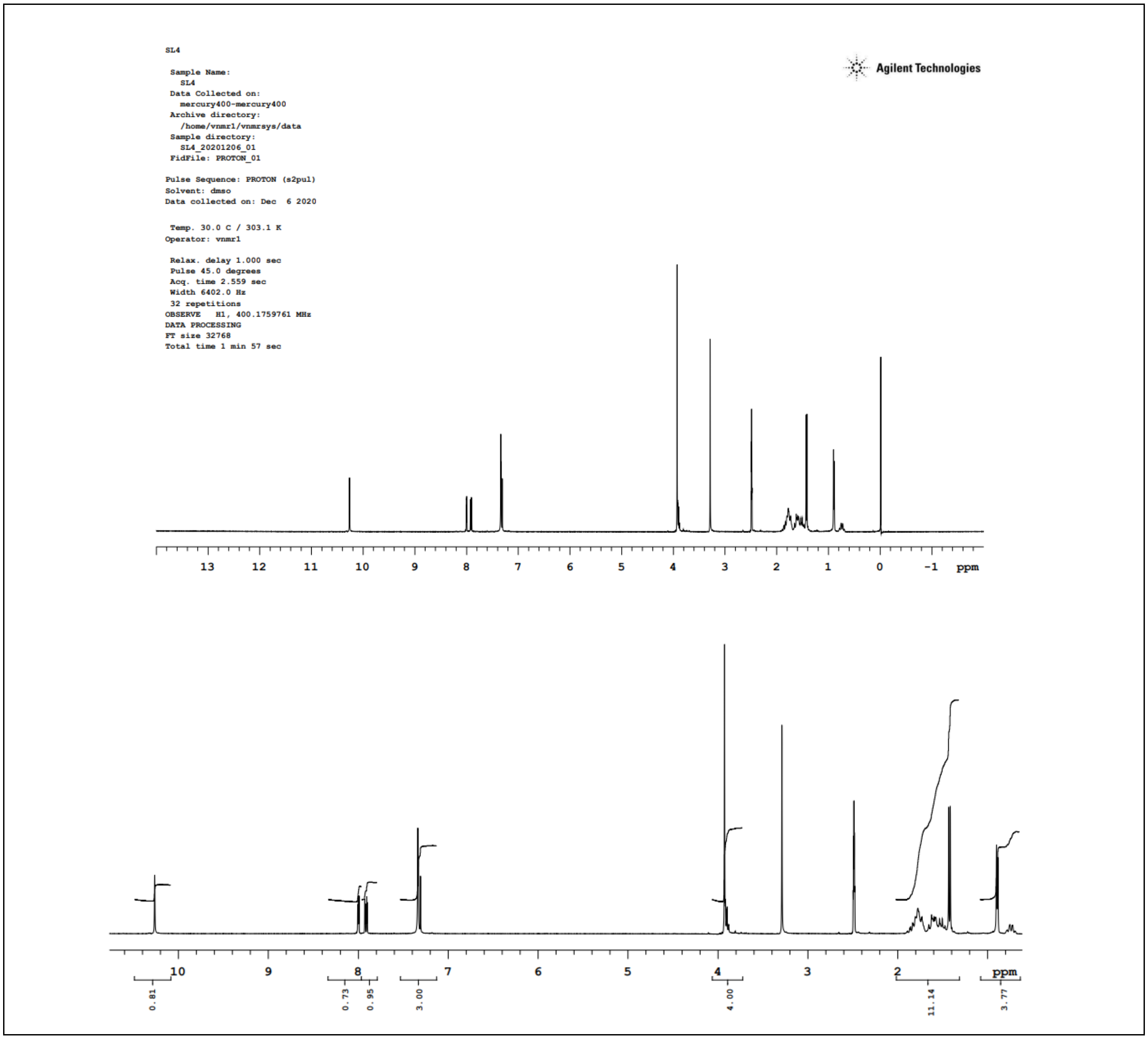

Figure S1: ^{1}H NMR spectrum of the compound **3a**

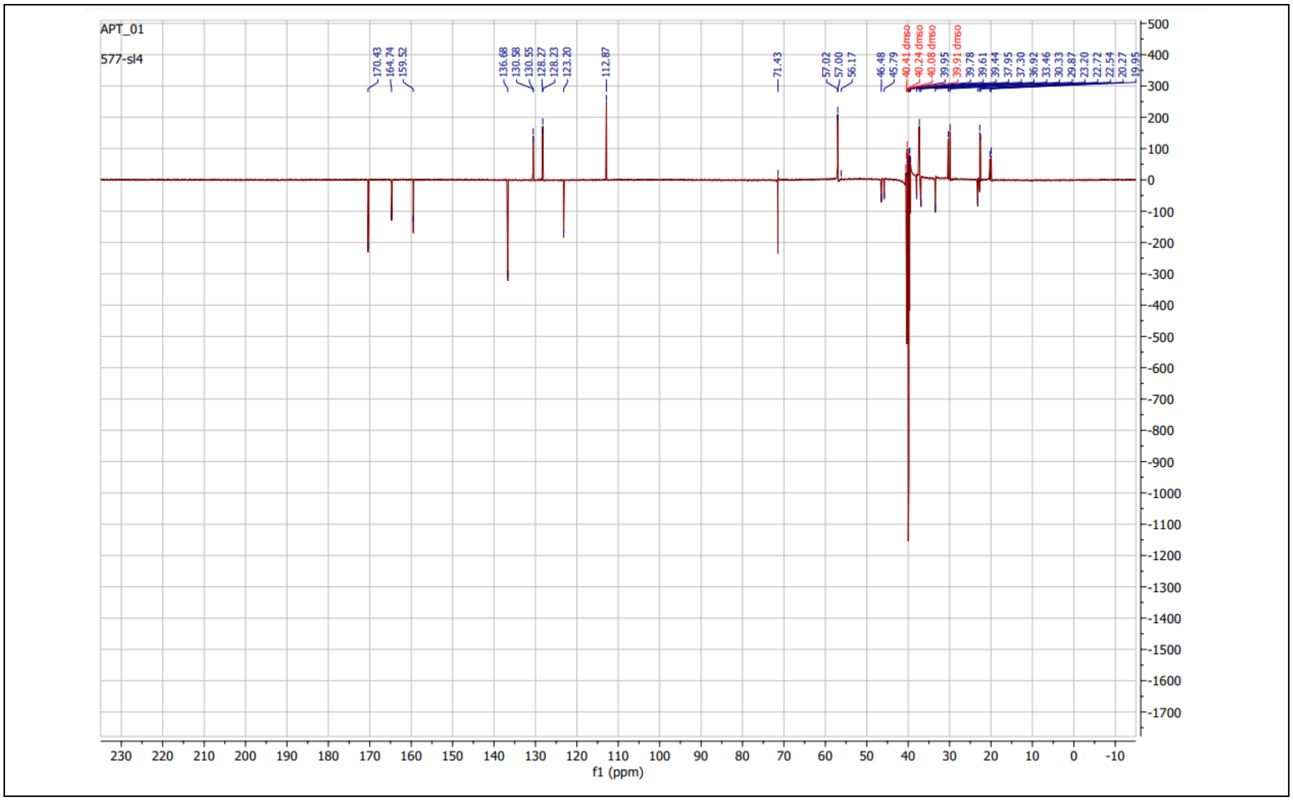

Figure S2: ^{13}C NMR spectrum of the compound **3a** (APT)

2-Methoxy-*N*-(2,8-dimethyl-3-oxo-1-thia-4-azaspiro[4.5]decan-4-yl)-4-sulfamoylbenzamide (3b)

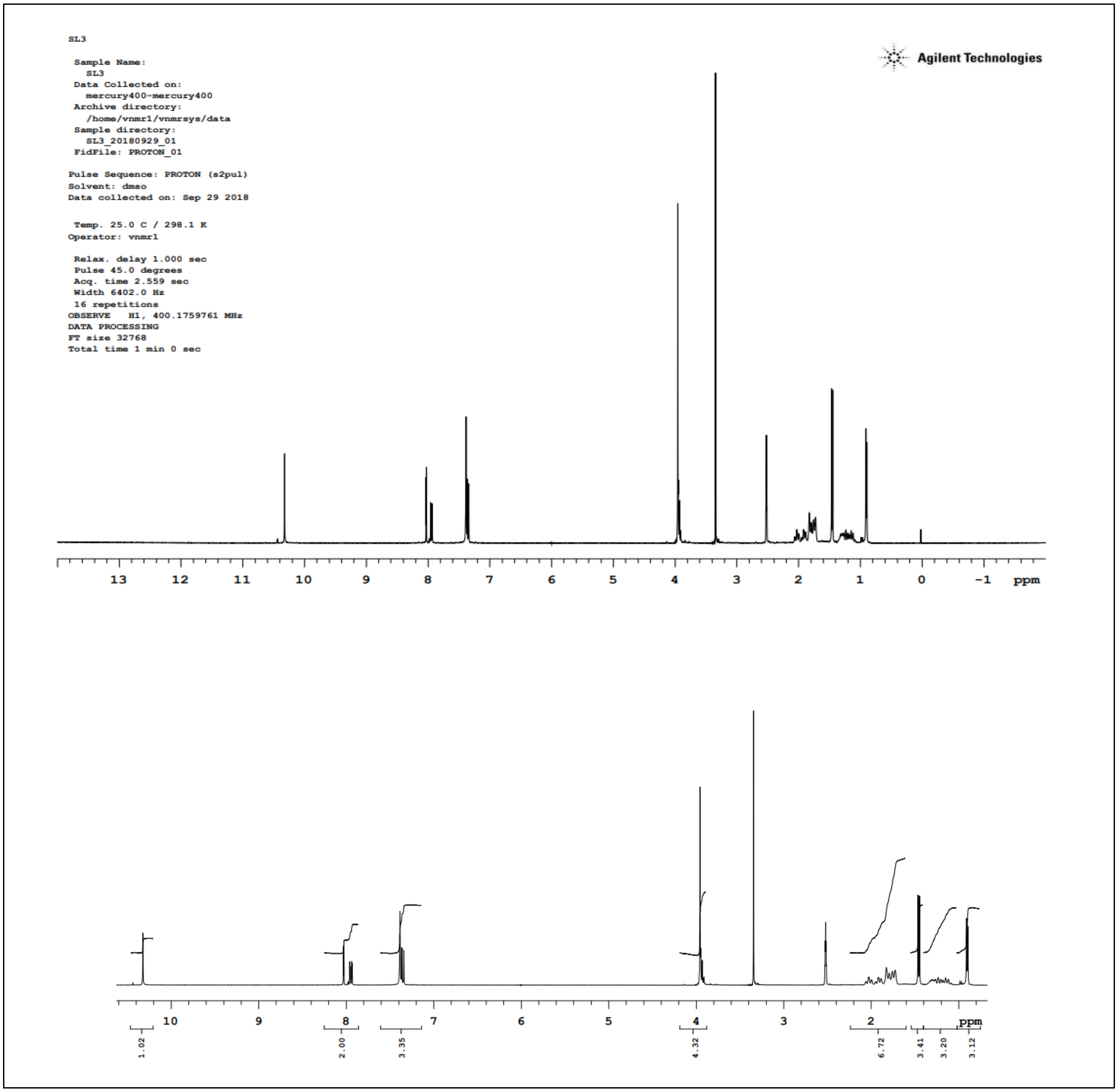

Figure S3: ^{1}H NMR spectrum of the compound **3b**

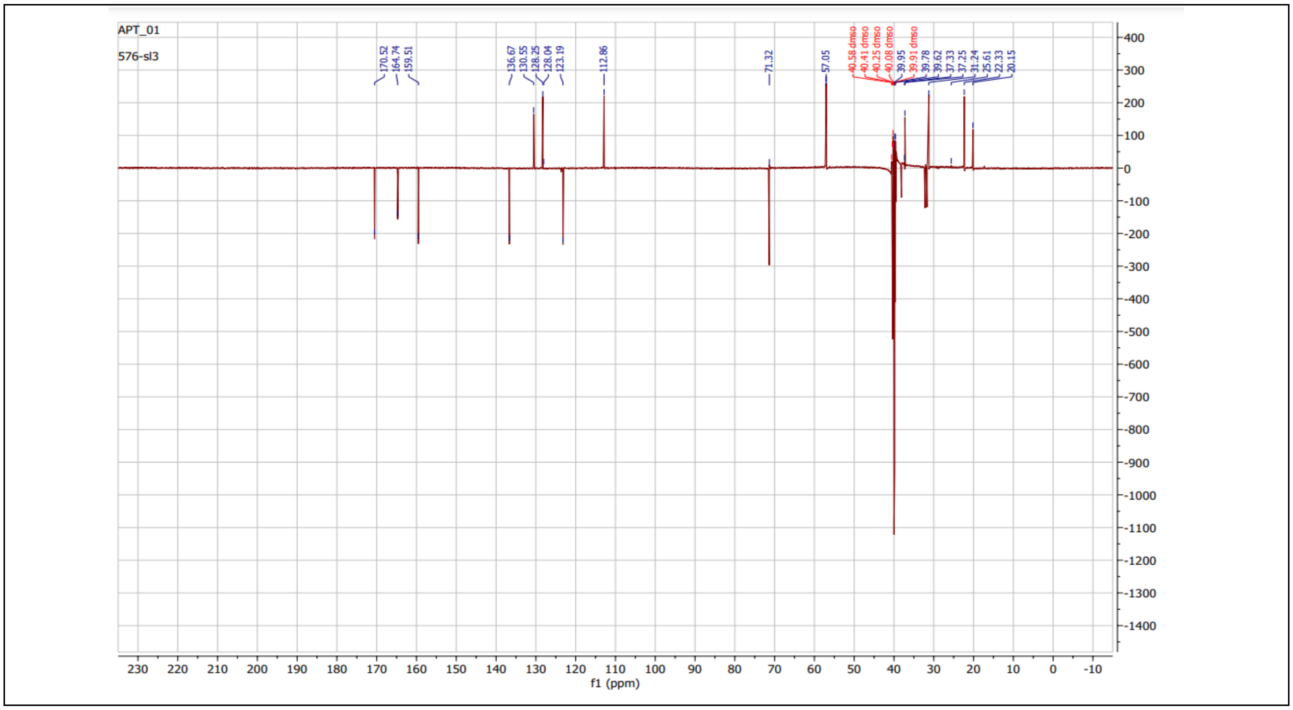

Figure S4: ^{13}C NMR spectrum of the compound **3b**

2-Methoxy-*N*-(8-ethyl-2-methyl-3-oxo-1-thia-4-azaspiro[4.5]decan-4-yl)-4-sulfamoylbenzamide (3c)

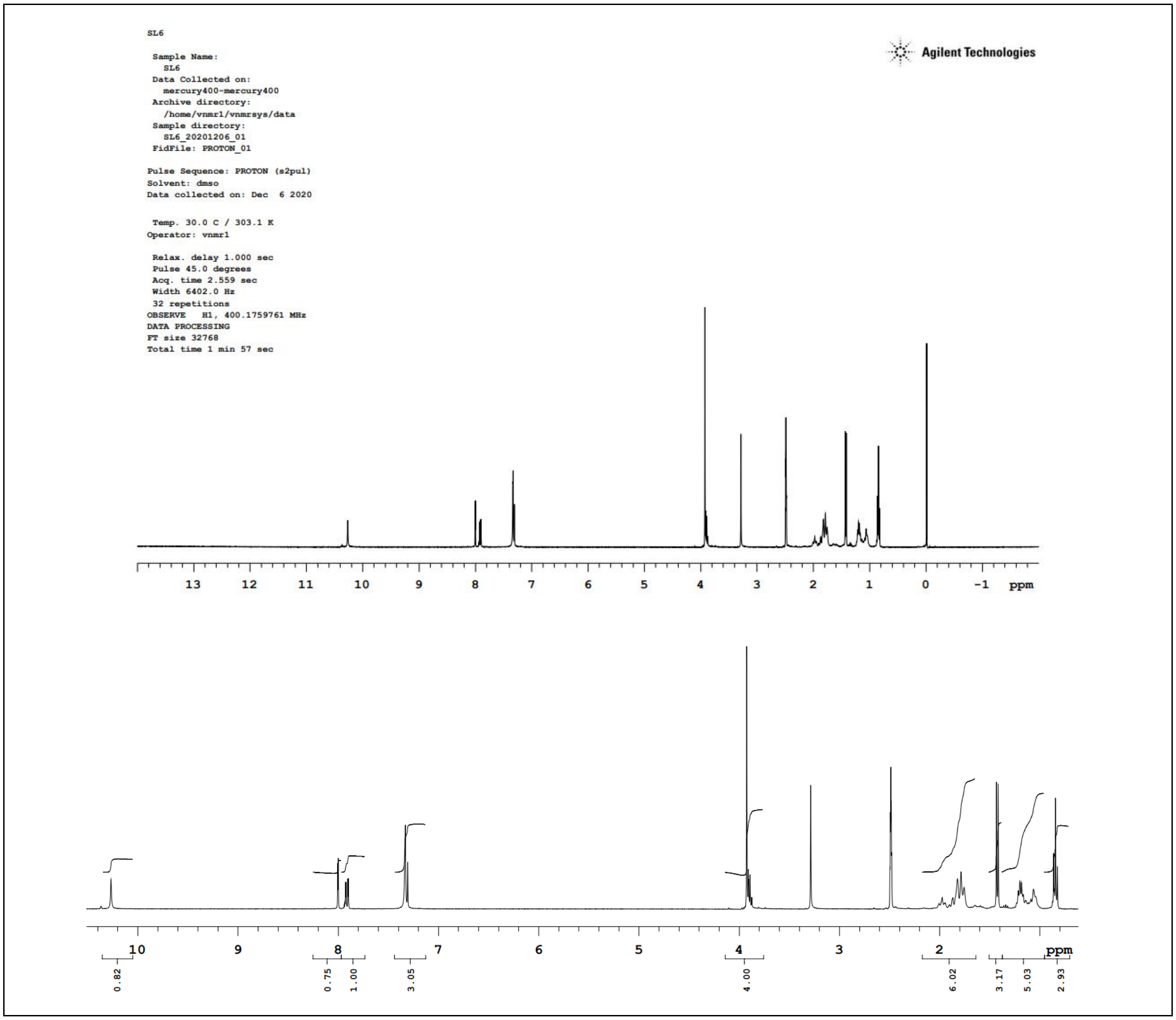

Figure S5: ^{1}H NMR spectrum of the compound **3c**

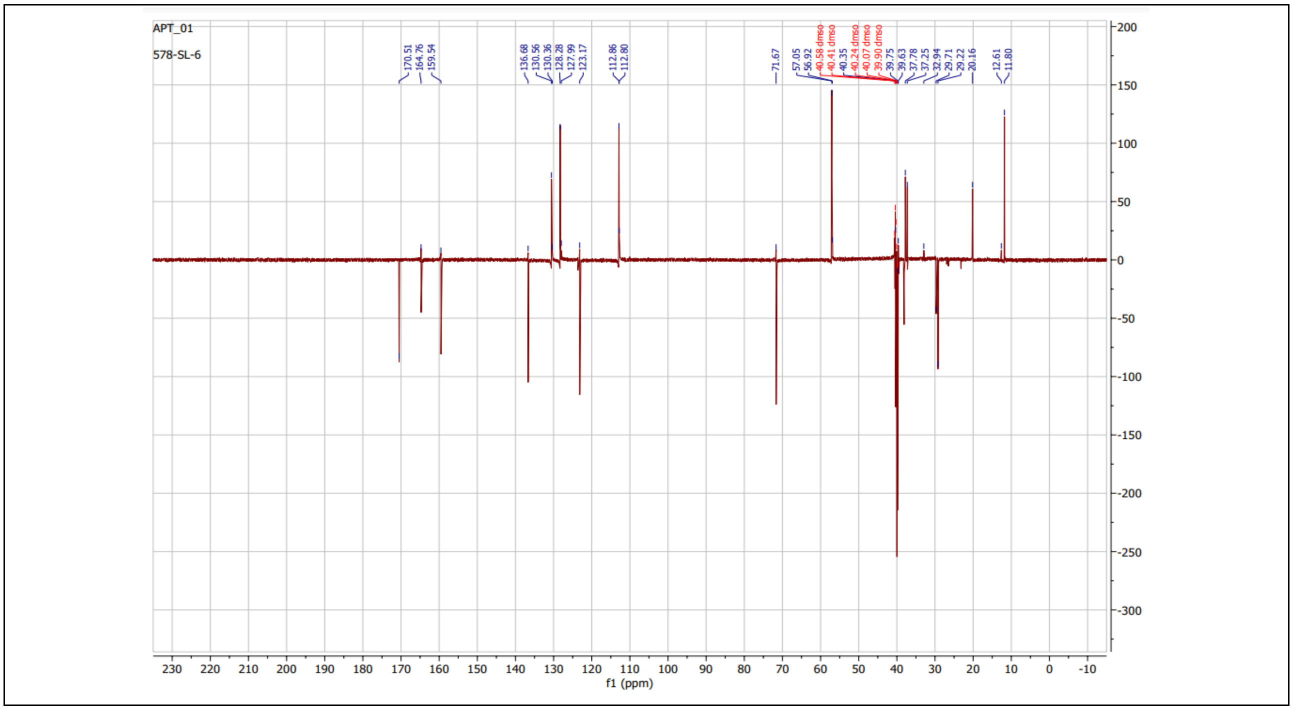

Figure S6: ^{13}C NMR spectrum of the compound **3c**

2-Methoxy-*N*-(2-methyl-3-oxo-8-propyl-1-thia-4-azaspiro[4.5]decan-4-yl)-4-sulfamoylbenzamide (3d)

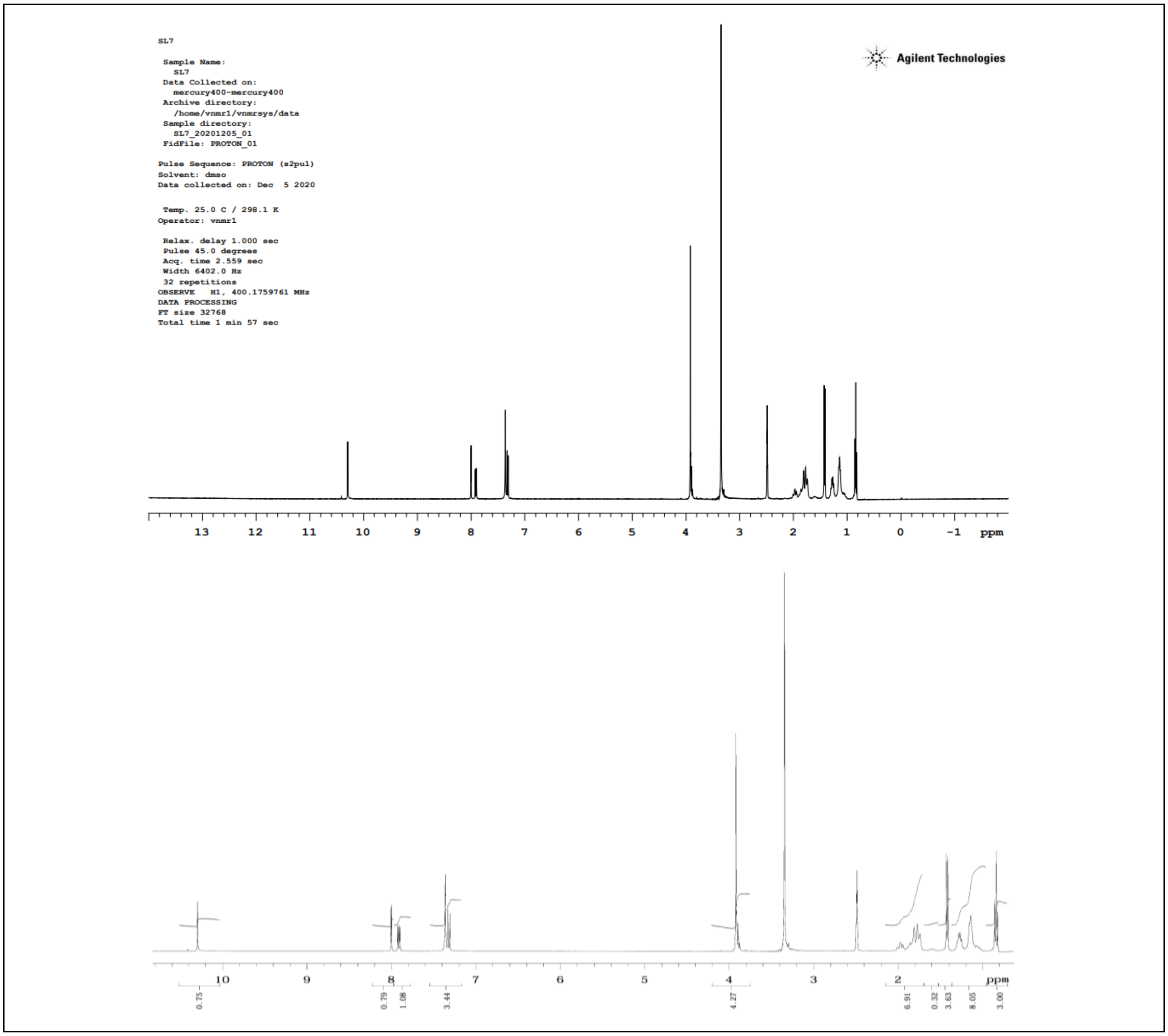

Figure S7: ^{1}H NMR spectrum of the compound **3d**

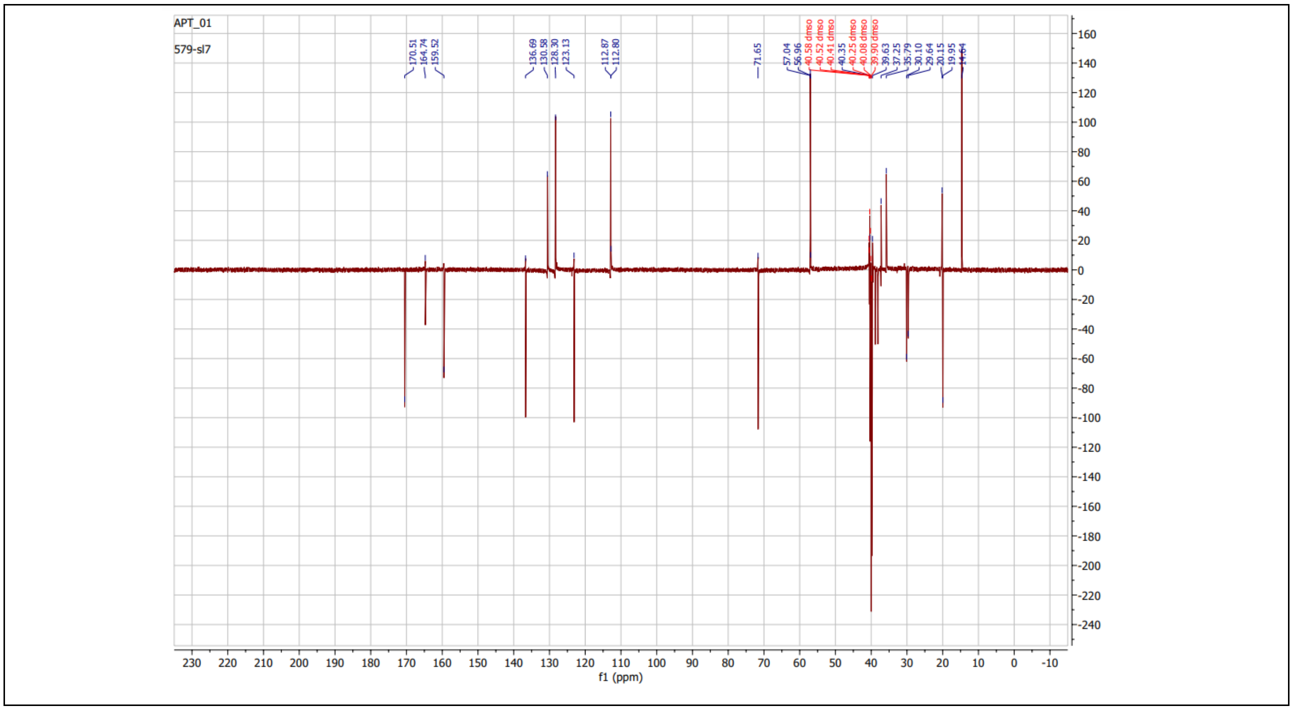

Figure S8: ^{13}C NMR spectrum of the compound **3d**

2-Methoxy-*N*-(2-methyl-3-oxo-8-*tert*-butyl-1-thia-4-azaspiro[4.5]decan-4-yl)-4-sulfamoylbenzamide (3e)

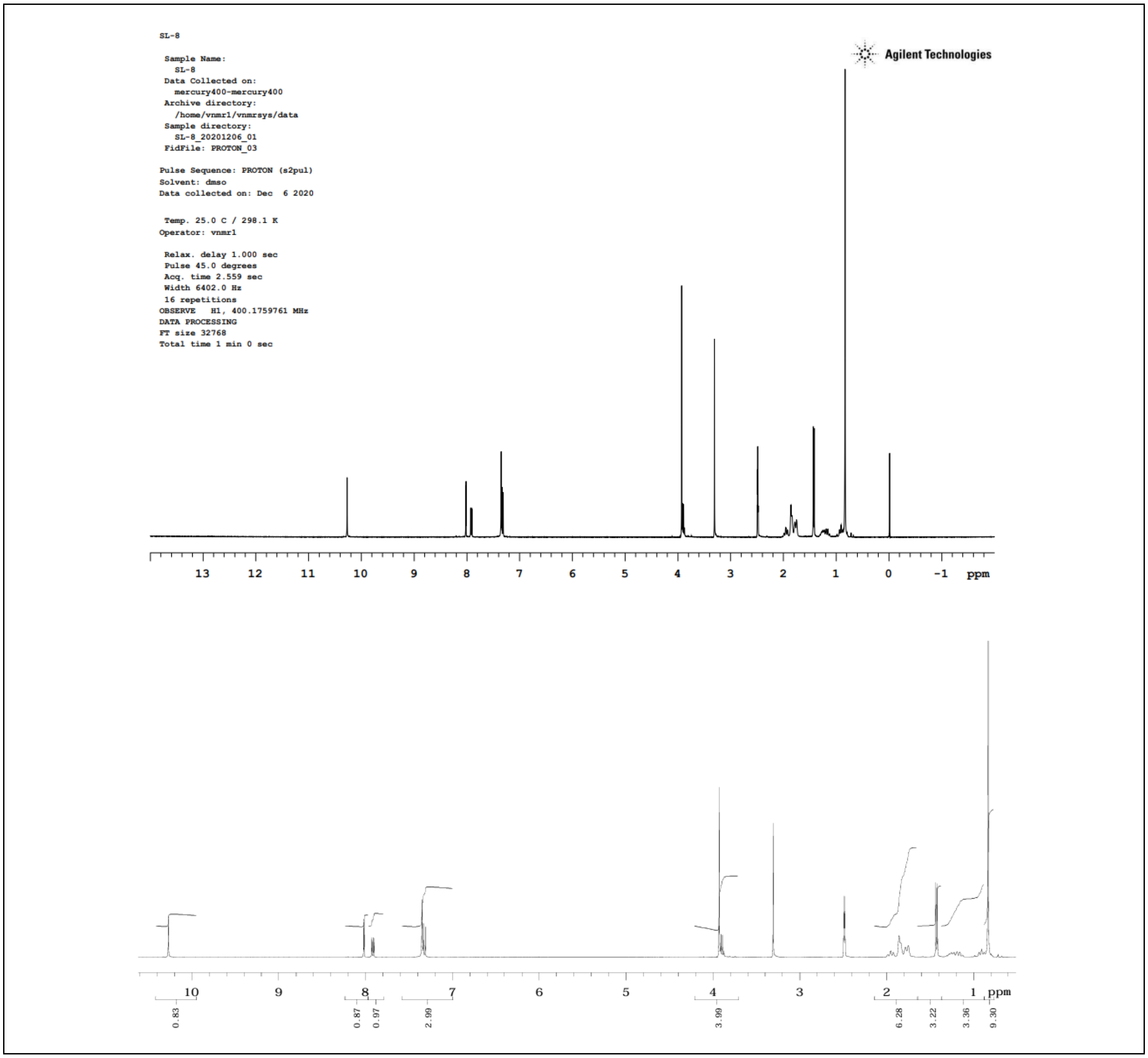

Figure S9: ^{1}H NMR spectrum of the compound **3e**

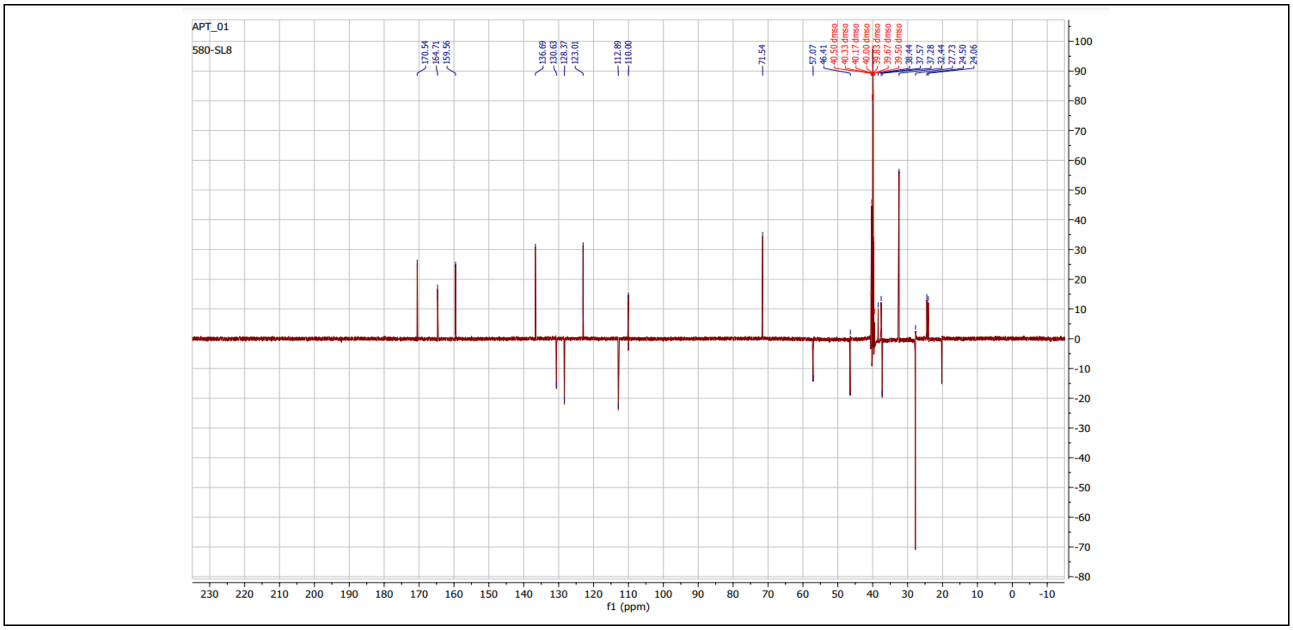

Figure S10: ^{13}C NMR spectrum of the compound **3e**

2-Methoxy-*N*-(2-methyl-3-oxo-8-*tert*-pentyl-1-thia-4-azaspiro[4.5]decan-4-yl)-4-sulfamoylbenzamide (3f)

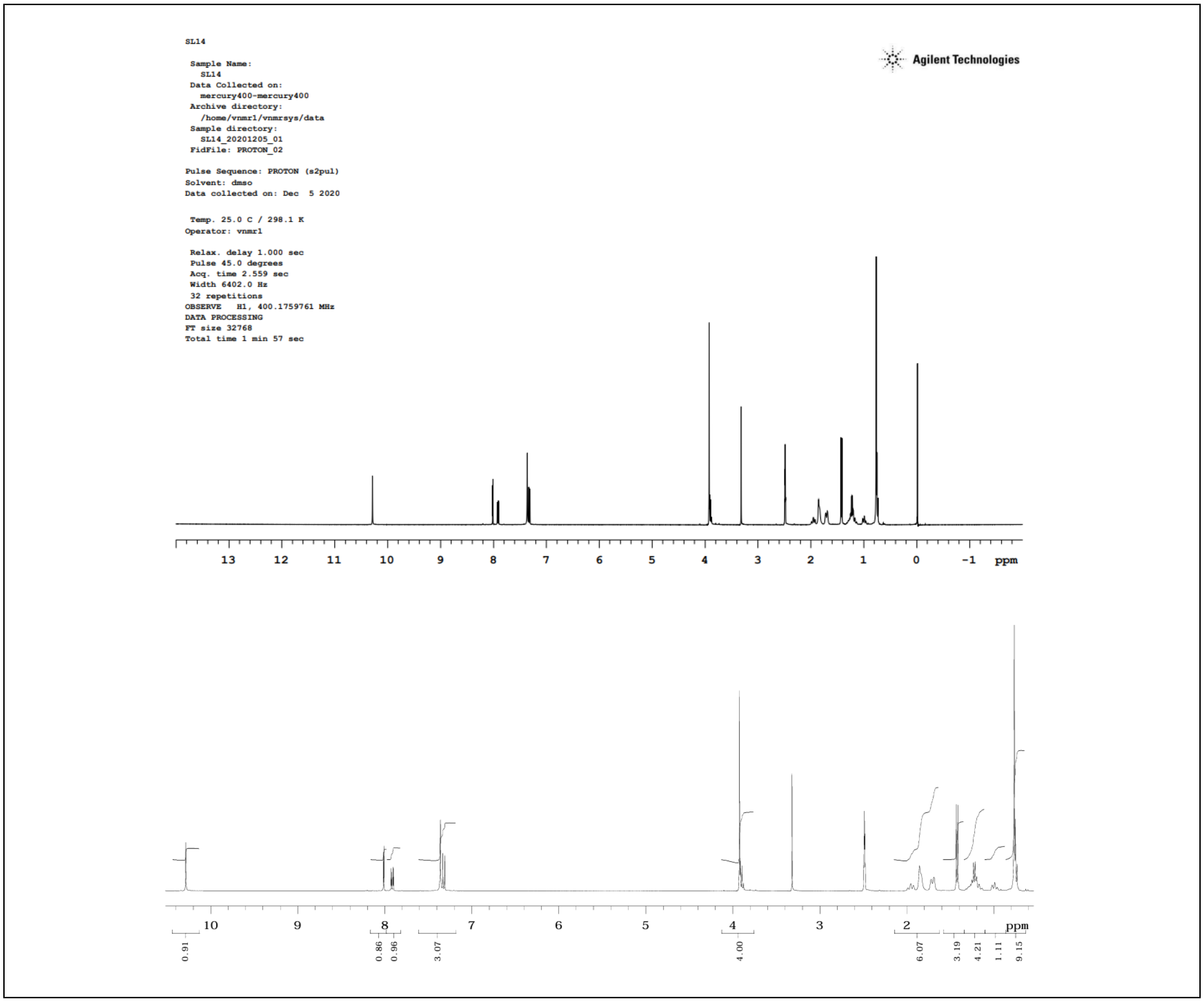

Figure S11: ^{1}H NMR spectrum of the compound **3f**

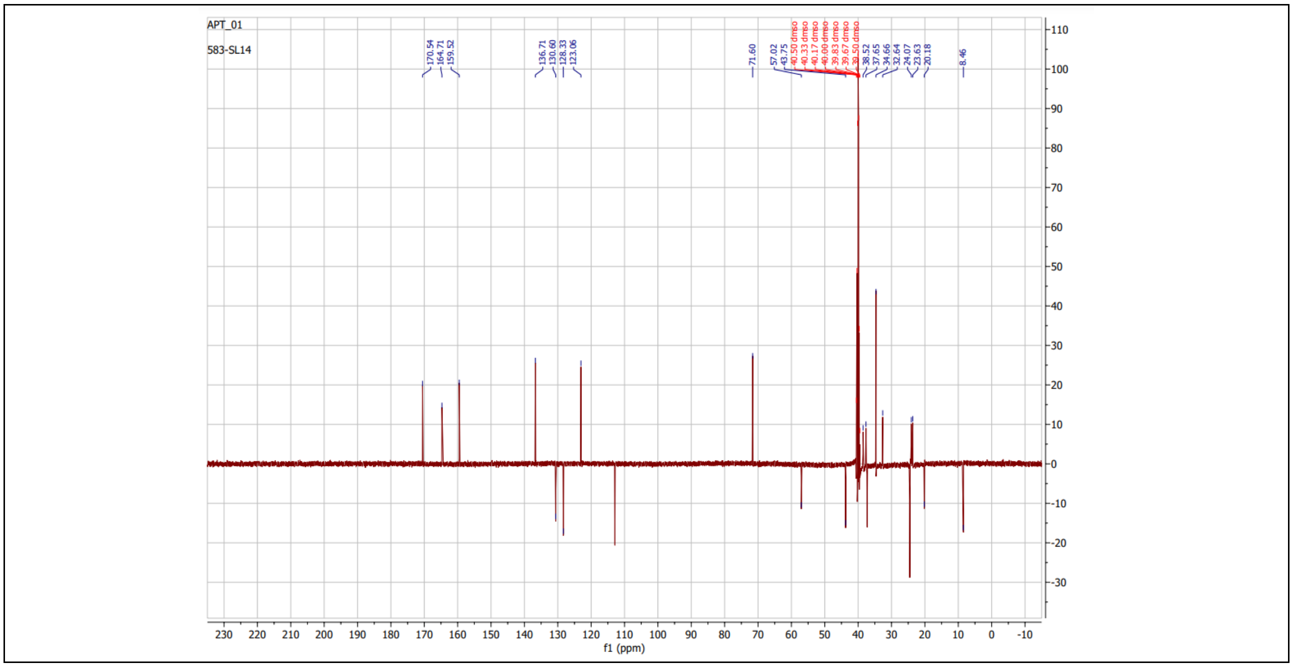

Figure S12: ^{13}C NMR spectrum of the compound **3f**

2-Methoxy-*N*-(2-methyl-3-oxo-1-thia-8-trifluoromethyl-4-azaspiro[4.5]decan-4-yl)-4-sulfamoylbenzamide (3g)

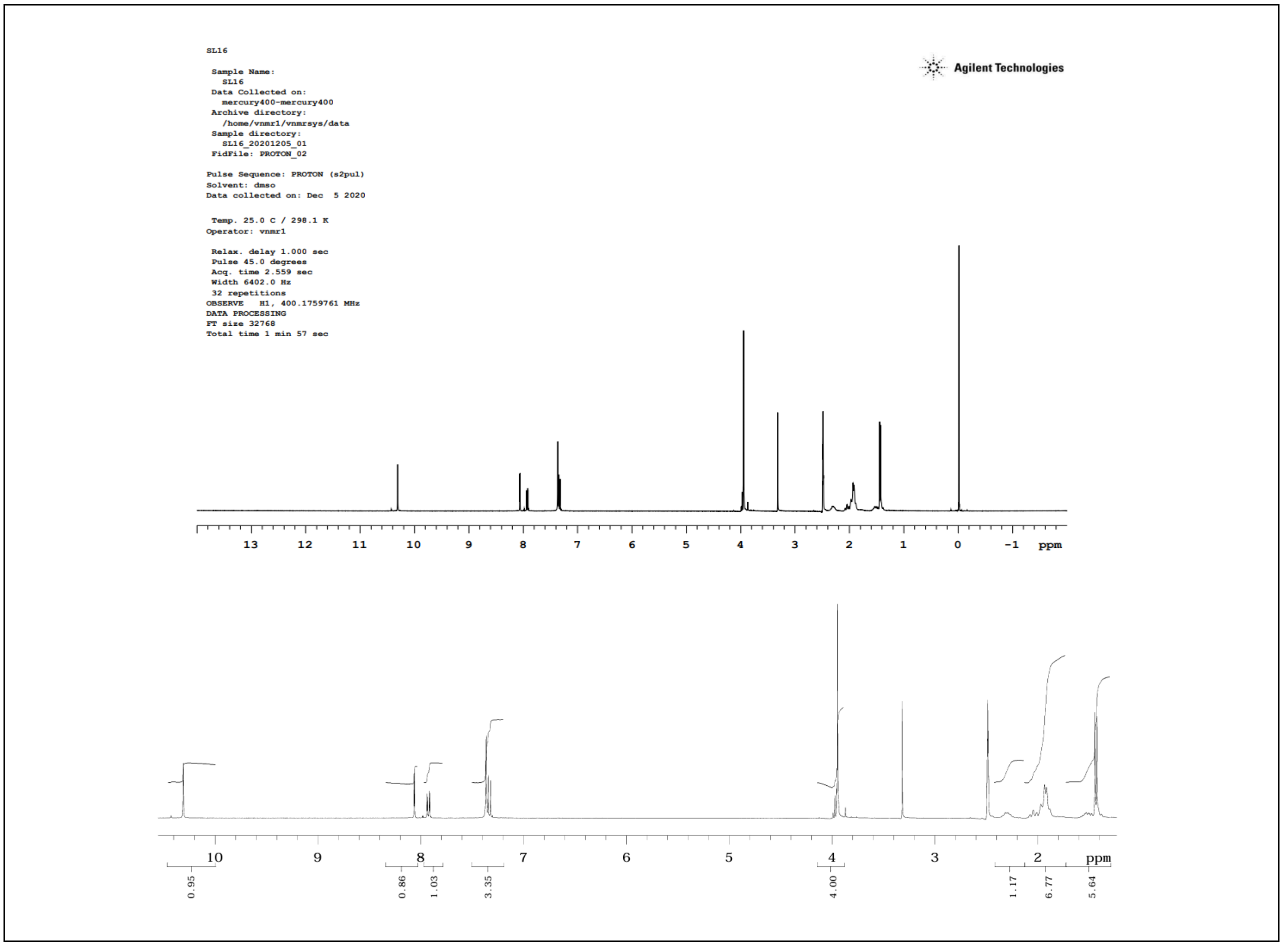

Figure S13: ^{1}H NMR spectrum of the compound **3g**

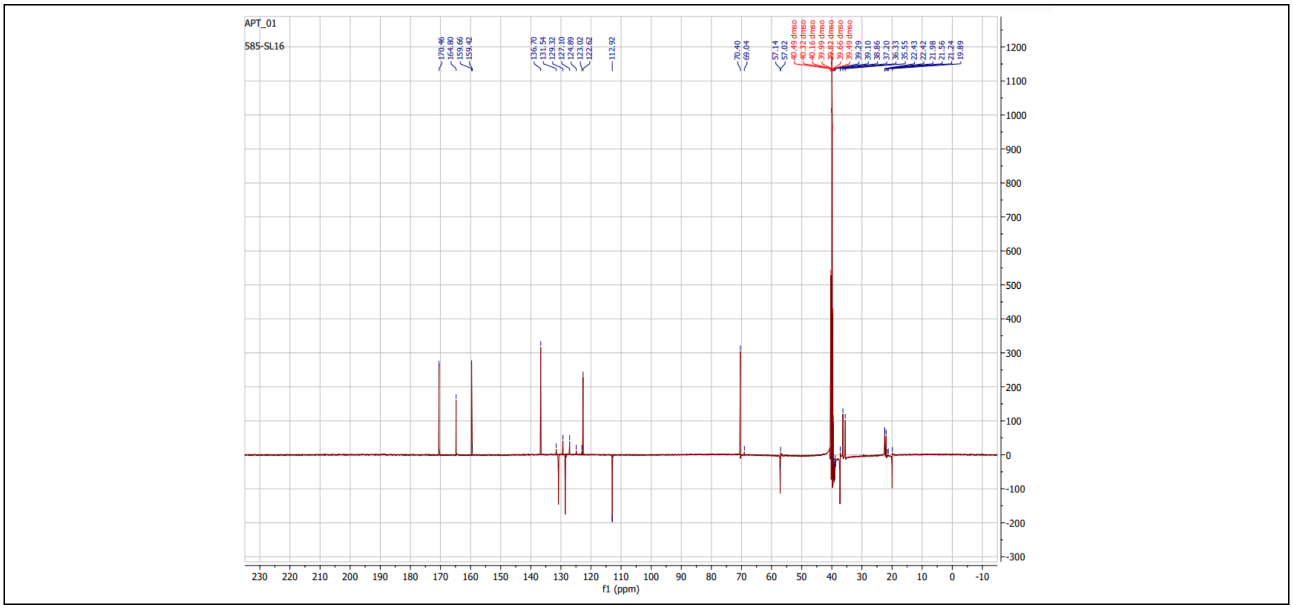

Figure S14: ^{13}C NMR spectrum of the compound **3g**

2-Methoxy-*N*-(2-methyl-3-oxo-8-phenyl-1-thia-4-azaspiro[4.5]decan-4-yl)-4-sulfamoylbenzamide (3h)

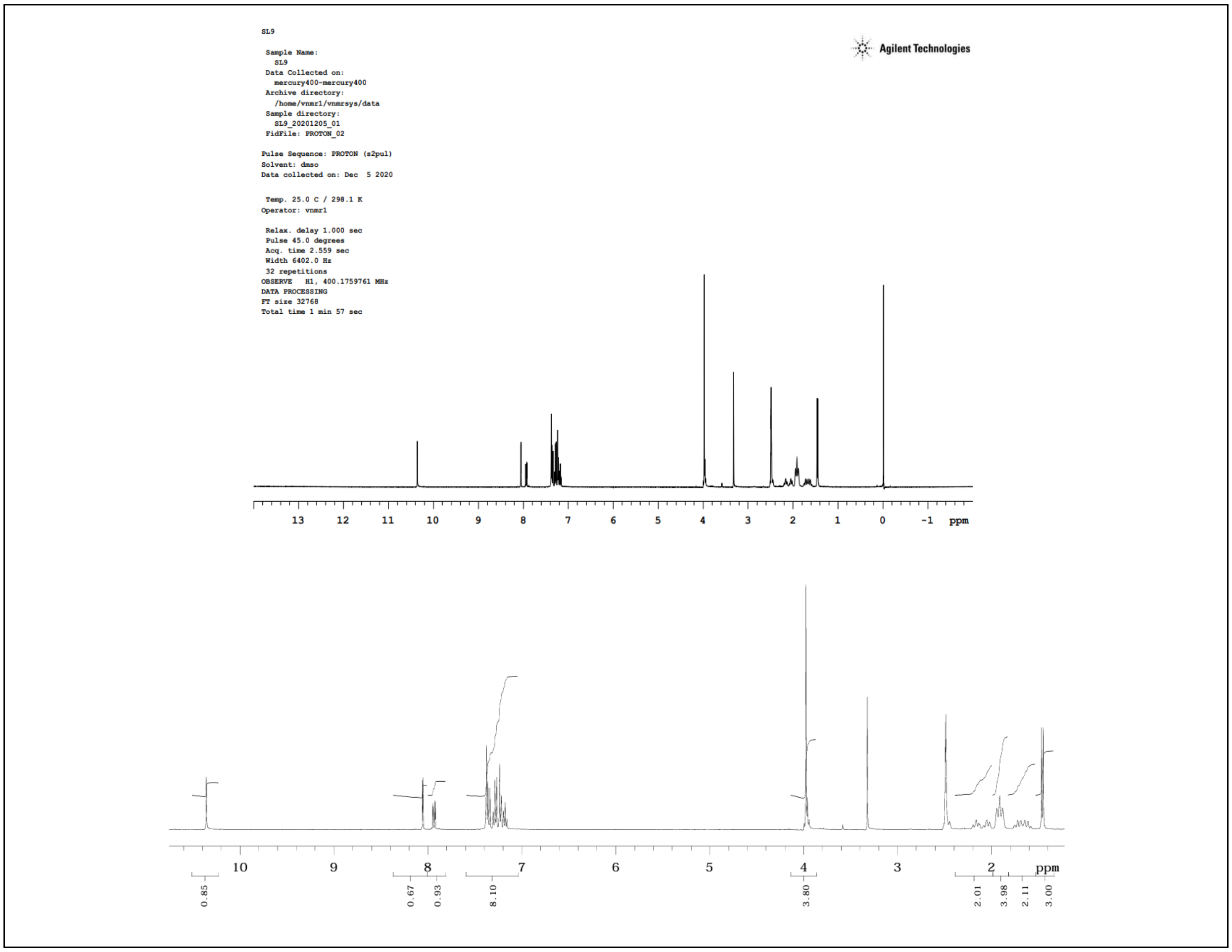

Figure S15: ^{1}H NMR spectrum of the compound **3h**

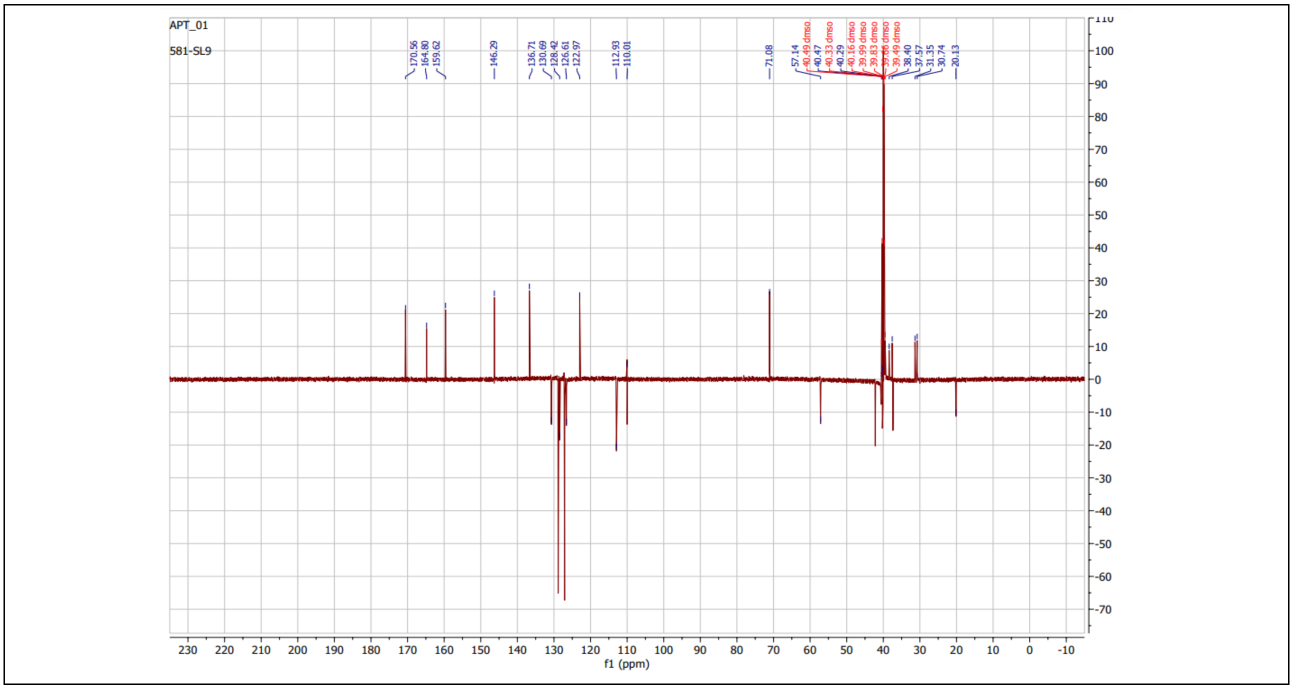

Figure S16: ^{13}C NMR spectrum of the compound **3h**

2-Methoxy-*N*-(8-cyano-2-methyl-3-oxo-8-phenyl-1-thia-4-azaspiro[4.5]decan-4-yl)-4-sulfamoylbenzamide (3i)

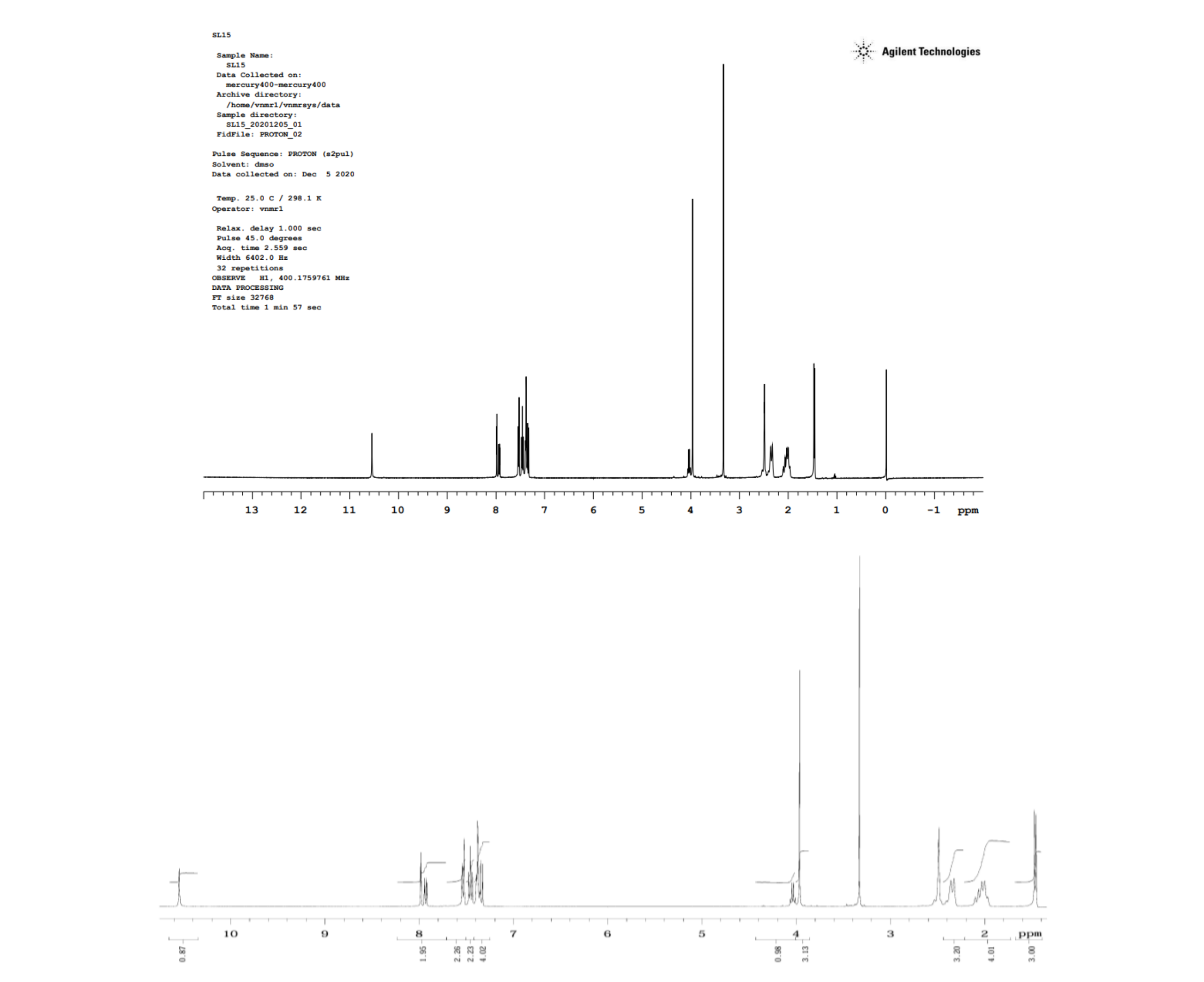

Figure S17: ^{1}H NMR spectrum of the compound **3i**

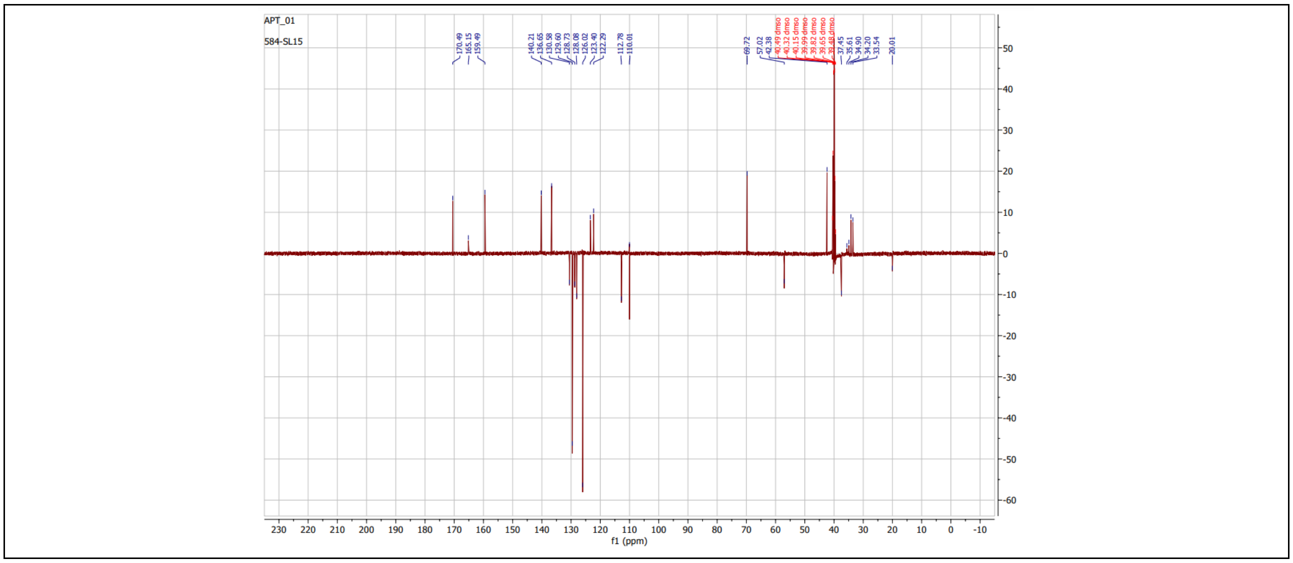

Figure S18: ^{13}C NMR spectrum of the compound **3i**

2-Methoxy-*N*-(8-acetamido-2-methyl-3-oxo-1-thia-4-azaspiro[4.5]decan-4-yl)-4-sulfamoylbenzamide (3j)

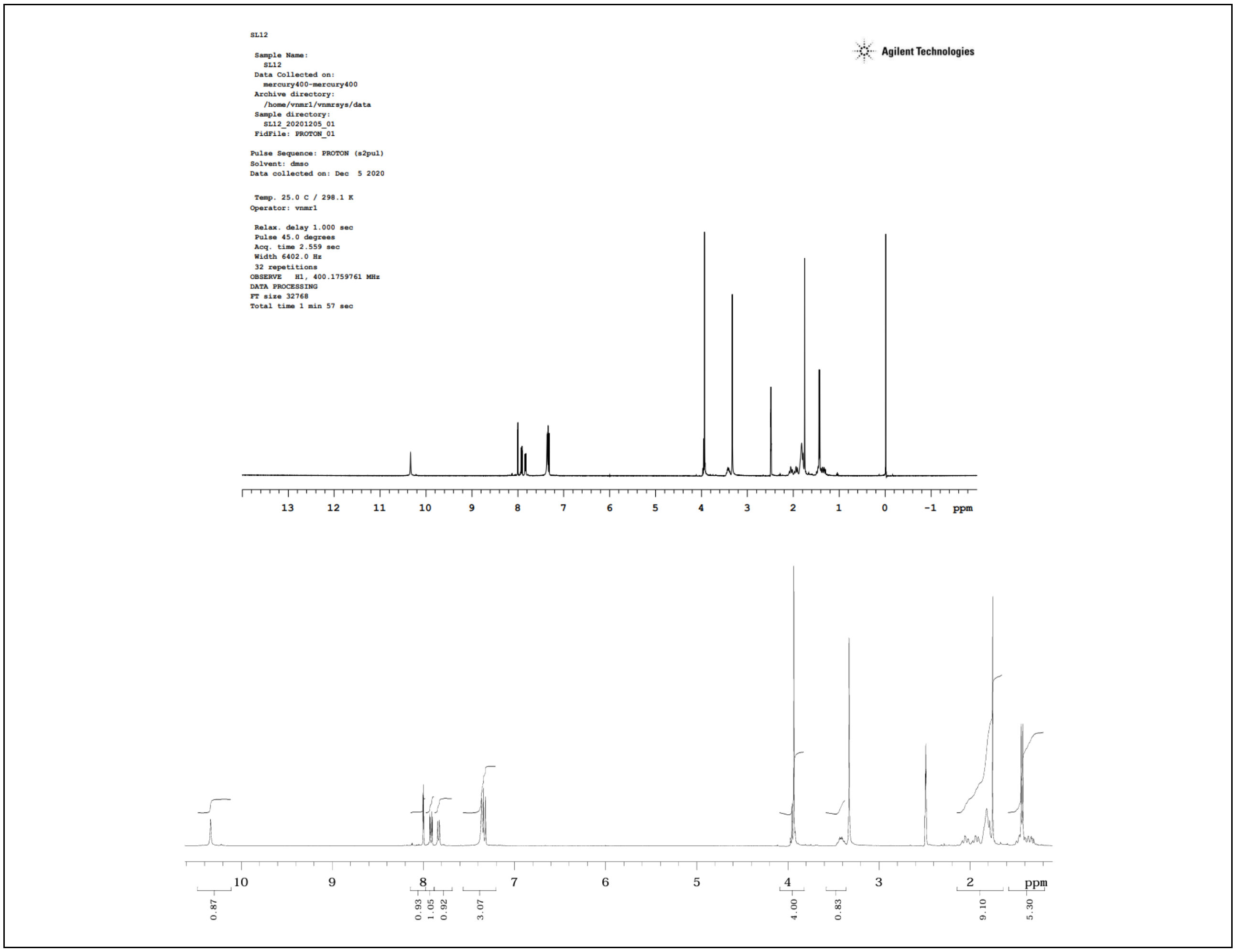

Figure S19: ^{1}H NMR spectrum of the compound **3j**

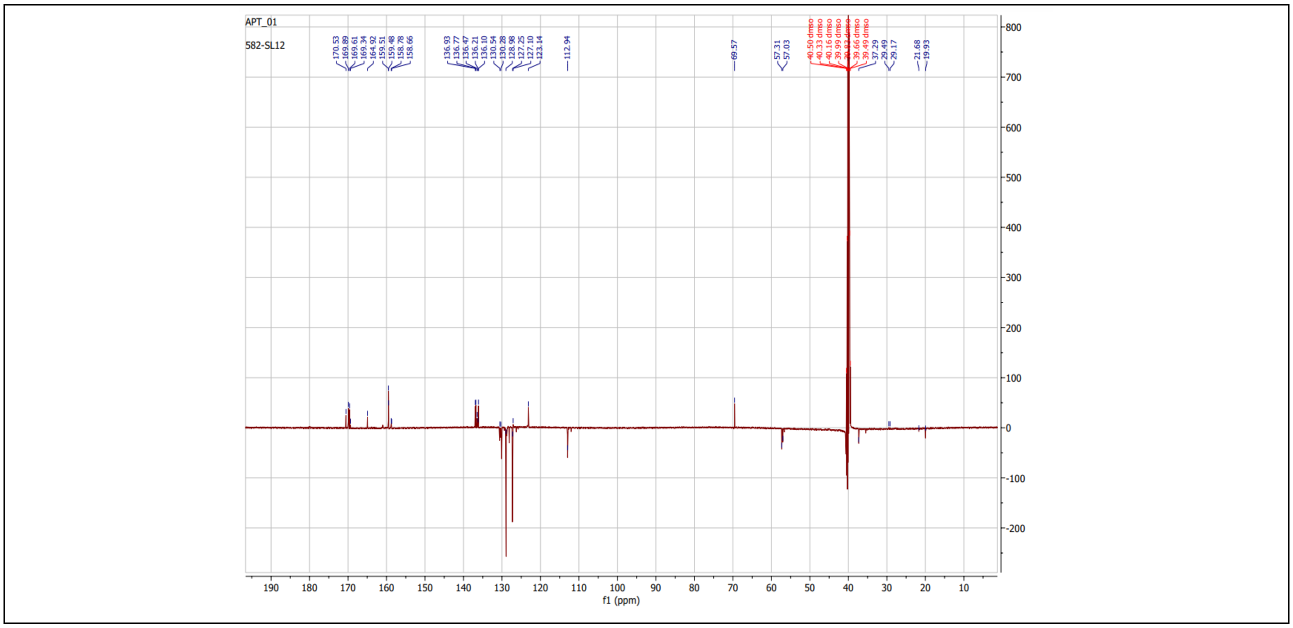

Figure S20: ^{13}C NMR spectrum of the compound **3j**
